# Machine learning-based dynamic mortality prediction after traumatic brain injury

**DOI:** 10.1038/s41598-019-53889-6

**Published:** 2019-11-27

**Authors:** Rahul Raj, Teemu Luostarinen, Eetu Pursiainen, Jussi P. Posti, Riikka S. K. Takala, Stepani Bendel, Teijo Konttila, Miikka Korja

**Affiliations:** 1 Department of Neurosurgery, Helsinki University Hospital and University of Helsinki, Topeliuksenkatu 5, PB 266, 00029 HUS Helsinki, Finland; 2 Division of Anesthesiology, Department of Anesthesiology, Intensive Care and Pain Medicine, Helsinki University Hospital and University of Helsinki, Topeliuksenkatu 5, PB 266, 00029 HUS Helsinki, Finland; 3 Data Scientist, Analytics and AI Development Services, HUS IT Management, Helsinki University Hospital, Haartmaninkatu 4, PB 340, 00029 HUS Helsinki, Finland; 4 Division of Clinical Neurosciences, Department of Neurosurgery, and Turku Brain Injury Centre, Turku University Hospital and University of Turku, Hämeentie 11, 20521 Turku, Finland; 5 Perioperative Services, Intensive Care Medicine and Pain Management, Turku University Hospital and University of Turku, Hämeentie 11, 20521 Turku, Finland; 6 Division of Intensive Care, Department of Anesthesiology, Intensive Care and Pain Medicine, Kuopio University Hospital, Puijonlaaksontie 2, 70210 Kuopio, Finland

**Keywords:** Prognostic markers, Brain injuries

## Abstract

Our aim was to create simple and largely scalable machine learning-based algorithms that could predict mortality in a real-time fashion during intensive care after traumatic brain injury. We performed an observational multicenter study including adult TBI patients that were monitored for intracranial pressure (ICP) for at least 24 h in three ICUs. We used machine learning-based logistic regression modeling to create two algorithms (based on ICP, mean arterial pressure [MAP], cerebral perfusion pressure [CPP] and Glasgow Coma Scale [GCS]) to predict 30-day mortality. We used a stratified cross-validation technique for internal validation. Of 472 included patients, 92 patients (19%) died within 30 days. Following cross-validation, the ICP-MAP-CPP algorithm’s area under the receiver operating characteristic curve (AUC) increased from 0.67 (95% confidence interval [CI] 0.60–0.74) on day 1 to 0.81 (95% CI 0.75–0.87) on day 5. The ICP-MAP-CPP-GCS algorithm’s AUC increased from 0.72 (95% CI 0.64–0.78) on day 1 to 0.84 (95% CI 0.78–0.90) on day 5. Algorithm misclassification was seen among patients undergoing decompressive craniectomy. In conclusion, we present a new concept of dynamic prognostication for patients with TBI treated in the ICU. Our simple algorithms, based on only three and four main variables, discriminated between survivors and non-survivors with accuracies up to 81% and 84%. These open-sourced simple algorithms can likely be further developed, also in low and middle-income countries.

## Introduction

In Europe and the U.S., more than 1.6 million people are hospitalized every year due to traumatic brain injury (TBI). Age-adjusted mortality rates are as high as 11.7/100,000 in Europe, 17.0/100,000 in the U.S. and even higher in low-and-middle income countries (LMIC), where the number of fatal traffic accidents seems to be increasing^1–3^.

Patients with moderate-to-severe TBIs are treated in the intensive care unit (ICU). Recent observational studies on ICU-treated TBI patients have reported mortality rates of approximately 30%^4,5^. The main purpose of intensive care after TBI is to mitigate the progression of secondary brain injury, by controlling intracranial pressure (ICP), cerebral perfusion pressure (CPP) and by maintaining cerebral homeostasis^6,7^. However, despite ICP and CPP being the cornerstones of TBI intensive care, none of the current prognostic models include these. In fact, current prognostic models for TBI are static in nature and are based upon simple variables that are assessed upon admission^8^. Of these static prediction models, which cannot react to changes in ICP and CPP, the IMPACT-TBI and the CRASH are among the most widely recognized and best validated MRC CRASH Trial Collaborators^9,10^. Still, these models are not applicable for the individual patient^11,12^.

ICU monitoring of TBI patients can generate hundreds of thousands of data points per patient every day. However, the human brain, is not capable of processing such amounts of data in decision-making processes. As a consequence, enormous amounts of important patient-specific data are wasted. Machine learning-based algorithms can capture non-linear feature correlations that are hard to detect by using classical statistical approaches. Thus, we hypothesized that it would be possible to design a machine learning-based algorithm that could capture dynamic changes in TBI prognosis, which occur during intensive care treatment. In more detail, the aim of this study was to develop a fully automated and objective, but still simple, dynamic algorithm that is based on ICP, mean arterial pressure (MAP) and CPP, since all these variables are routinely measured in most ICUs. Furthermore, we aimed to develop a second algorithm, including components of the widely used Glasgow Coma Scale (GCS)^13^.

## Methods

### Ethical issues

The research committees of Helsinki University Hospital (HUS/356/2017; HUS/125/2018), Kuopio University Hospital (TJ 280/2014, 507T013), and Turku University Hospital (TP2/008/18) approved the study and waived the need for informed consent. We adhered to the Transparent Reporting of a multivariable prediction model for Individual Prognosis Or Diagnosis (TRIPOD) statement (Note 1 in the Electronic Supplementary Material)^14^.

### Study design and patients

We conducted a retrospective multicenter study including consecutive adult TBI patients (16 years or older) who were admitted (within 24 h of trauma) to one of the three tertiary academic ICUs (Helsinki University Hospital [during 2010–2017], Kuopio University Hospital [during 2004–2013], and Turku University Hospital [during 2003–2013]). We only included patients that underwent ICP monitoring for more than 24 h. Thus, if ICP monitoring was stopped due to death or deemed unnecessary within 24 h we did not include these patients.

### Treatment and monitoring protocols

All included centers adhered to the most up-to-date version of Brain Trauma Foundation guidelines or to the European Brain Injury Consortium guidelines^15–18^. All centers used parenchymal probes or ventricular catheters to measure ICP (Codman DePuy Synthes, Johnson & Johnson, New Brunswick, NJ, US or Raumedic, Raumedic AG, Helmbrechts, Germany) and targeted ICP below 20 mmHg. All centers monitored invasive intra-arterial blood pressure (BD Cabarith PMSET IDT-XX, Singapore, or similar devices), targeted CPP at 60–70 mmHg and used CPP-guided treatment in case of intracranial hypertension with disturbed autoregulation. All centers routinely elevated the head of the patient to 30 degrees and maintained physiological body temperature (paracetamol or low dose diclofenac infusion, external of intravascular cooling). In addition to continuous sedation, treatment options to lower ICP included osmotherapy (predominantly hypertonic saline), normothermia or mild hypothermia, hyperventilation, external ventricular cerebrospinal fluid drainage, use of barbiturates and decompressive craniectomy.

### Data collection and preprocessing

#### Baseline characteristics

We obtained patient baseline characteristics according to the International Mission for Prognosis and Analysis of Clinical Trials in TBI (IMPACT-TBI^10^) through electronic health records (including emergency medical service reports, hospital records, surgical reports, laboratory reports and picture achieving and communication systems). We assessed admission GCS score and if the patient were intubated and/or sedated we used the best pre-intubation and post-resuscitation GCS score. We defined hypoxia as a documented oxygen saturation < 90% and hypotension as a measured systolic blood pressure < 90 mmHg during the pre-hospital period^19^. We classified head CTs according to the Marshall CT classification^20^. We obtained interventions-related data of external ventricular drainage (EVD), craniotomies for mass lesions and decompressive craniectomies (DC). We defined a DC as primary if the patient underwent emergency DC and secondary if the DC was performed due to intractable ICP, regardless of previous mass lesion evacuation.

#### ICU variables

We collected ICP, MAP, CPP (difference in MAP and ICP) and GCS score data from electronic databases up to five days (“PICIS Critical Care Suite”, PICIS Clinical Solutions, Barcelona, Spain and “Centricity Critical Care Clinisoft”, GE Healthcare, Ill, USA). We collected ICP (9 Hz), MAP (22 Hz) and CPP (9 Hz) in 1 to 5-minute intervals (median values) and rounded them to the nearest 5-minute time resolution. We excluded extreme measurement values (ICP > 100 mmHg or < 0 mmHg, MAP > 150 mmHg or < 20 mmHg). Of the GCS components, we extracted the motor and eye components. We did not use the verbal component as all patients were intubated and mechanically ventilated at some point. In all participating ICUs, skilled neurointensive nurses tested the GCS components following the wake-up test, in which sedation is transiently ceased^21^.

### Outcome

We used 30-day all-cause mortality as the primary outcome. We calculated time to death from time of first hospital admission. We obtained the dates of death from the Finnish Population Register (available for all patients).

### Dynamic algorithms

We aimed to develop a fully automated and objective dynamic algorithm based upon ICP, MAP and CPP to predict 30-day mortality. Further, we also aimed to develop a second algorithm including the motor and eye response components of the GCS. We designed both algorithms to give the first prediction after 24 h and new predictions every 8 h up to 120 h. For patients dying within the first 5 days, we excluded the last 12 h prior to death in order to avoid bias caused by treatment withdrawal. Thus, patients dying within 36 h were not included.

We started by generating dynamic features for ICP, MAP, CPP, motor response and eye response. Features were designed as means from the first 24 h time-window (begin), means from the last 8 h time-window (end), linear trend coefficients from the last time-window (coef), minimum values from the last time-window (min), maximum values from the last time-window (max), means of differences from the last time-window (diff), variances from the last time-window (var) and mean values from the last time-window (avg). For ICP, we designed features capturing the percentage of measured data-points being higher than 20 mmHg (ht20) and lower than 10 mmHg (lt10) in the last time-window. For MAP, we designed features capturing the percentage of measured data-points being higher than 120 mmHg (ht120) as a measure of severe arterial hypertension. Furthermore, we designed features capturing the trends of the most extreme values in terms of the highest 90^th^ percentile (q90) and the lowest 10^th^ percentile (q10). Finally, 54 features (+age) were considered for the ICP-CPP-MAP model and 74 features (+age) were considered for the ICP-CPP-MAP-GCS model (Note 2 in the Electronic Supplementary Material). In order to avoid overfitting, we used recursive elimination (scikit-learn: sklearn.feature_selection.RFECV) to select the optimal number of features (Note 2 in the Electronic Supplementary Material). The recursive feature elimination included data from the whole five-day time-period. Following recursive feature elimination, the chosen features were included in the algorithms. In the algorithms, we kept the features’ coefficients constant, but the features’ values were calculated in rolling time-windows (every 4 h for ICP, MAP, CPP and 24 h for GCS score components). All feature values were normalized to values between 0 and 1.

In order to make the algorithms as generalizable and simple as possible, we did not imputate any missing values. Since we used 5-minute median values as described above, all variables in the algorithms can be calculated even if some values are missing in the preceding time-window. If values were completely missing for the time window (4 h for ICP, MAP, CPP and 24 h for GCS), we excluded the specific patient from the specific time window-based estimate.

### Statistical analysis

We conducted all statistical analyses were conducted using SPSS Statistics for Windows, version 24.0, released 2016 (IBM Corp, Armonk, NY, USA), Stata version 14 (StataCorp, College Station, TX) and Google Cloud Platform (GCP). We conducted the statistical analyses in collaboration with a Google Cloud Platform partner, Qvik Ltd (Helsinki, Finland). The GCP codes are available in Notes 3 and 4 in the Electronic Supplementary Material.

We tested for differences in categorical data between survivors and non-survivors using a two-sided χ^2^ test. We tested continuous data for skewness. We tested for differences in skewed data between survivors and non-survivors using a non-parametric Mann-Whitney U test and for normally distributed data using a t-test. We used a logistic regression approach using the scikit-learn package for the creation of the dynamic algorithms.

To assess performance of the dynamic algorithms, we calculated the area under the receiver operating characteristic curve (AUC) as a function of time, in order to assess how the algorithms performed at different time points. For the AUC analyses, we used a repeated five-fold stratified cross validation technique (20 repetitions) to reduce overfitting, as the expected number of deaths was notably lower than survivors^22^. We further assessed the number of false positives (i.e. patients that that were predicted to have a fatal outcome but survived) and the number of false negatives (i.e. patients that were predicted to survive but had a fatal outcome). In more detail, if the last given probability of 30-day mortality was over 50% and the patient survived, we classified the case as a false positive. We set both dynamic algorithms’ thresholds for predicting death at 50% in order to minimize the number of false positives. We conducted a descriptive analysis looking at the false positives generated from the ICP-MAP-CPP algorithm. In order to estimate the performance of the algorithms, we compared their estimates of the risk of 30-day mortality to the widely used and validated IMPACT-TBI lab -based model (referred to as IMPACT-TBI model) by fitting all IMPACT parameters in a logistic regression model and assessing its AUC and calibration^10,23^.

### Statistical analyses

Conducted by data scientists Eetu Pursiainen and Teijo Konttila with assistance of Mikko Kemppainen (data scientist) and Olli Paakkunainen (software engineer) from Qvik (qvik.com).

## Results

### Patient characteristics

**Figure 1:**
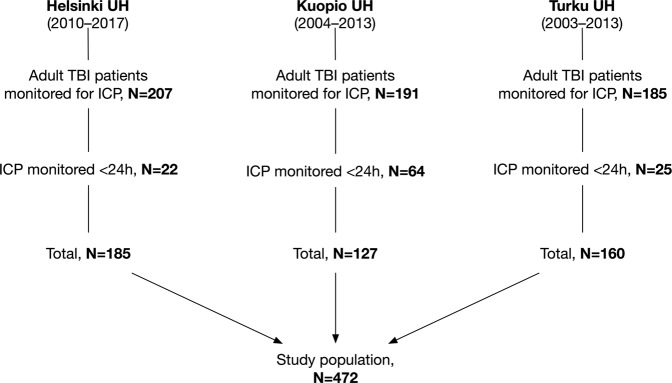
Flow chart. *Abbreviations*: HUS, Helsinki University Hospital; KUH, Kuopio University Hospital; TUH, Turku University Hospital; ICP, intracranial pressure; TBI, traumatic brain injury.

A total of 472 patients were included (Fig. 1). After excluding the last 12 h of monitored data for patients dying within the first five days (N = 28), the mean count of 5-minute median ICP values was 1,080 per patient and the mean count of 5-minute median MAP values was 1,308 per patient. Thus, the mean time of ICP monitoring was 90 h per patient (cumulatively 42,620 h) and the mean time of MAP monitoring was 109 h per patient (cumulatively 51,534 h). The mean number of missing values per patient were as follows: 70 values for ICP (SD 117), 78 values for MAP (SD 130), 70 values for CPP (SD 117).

**Figure 2:**
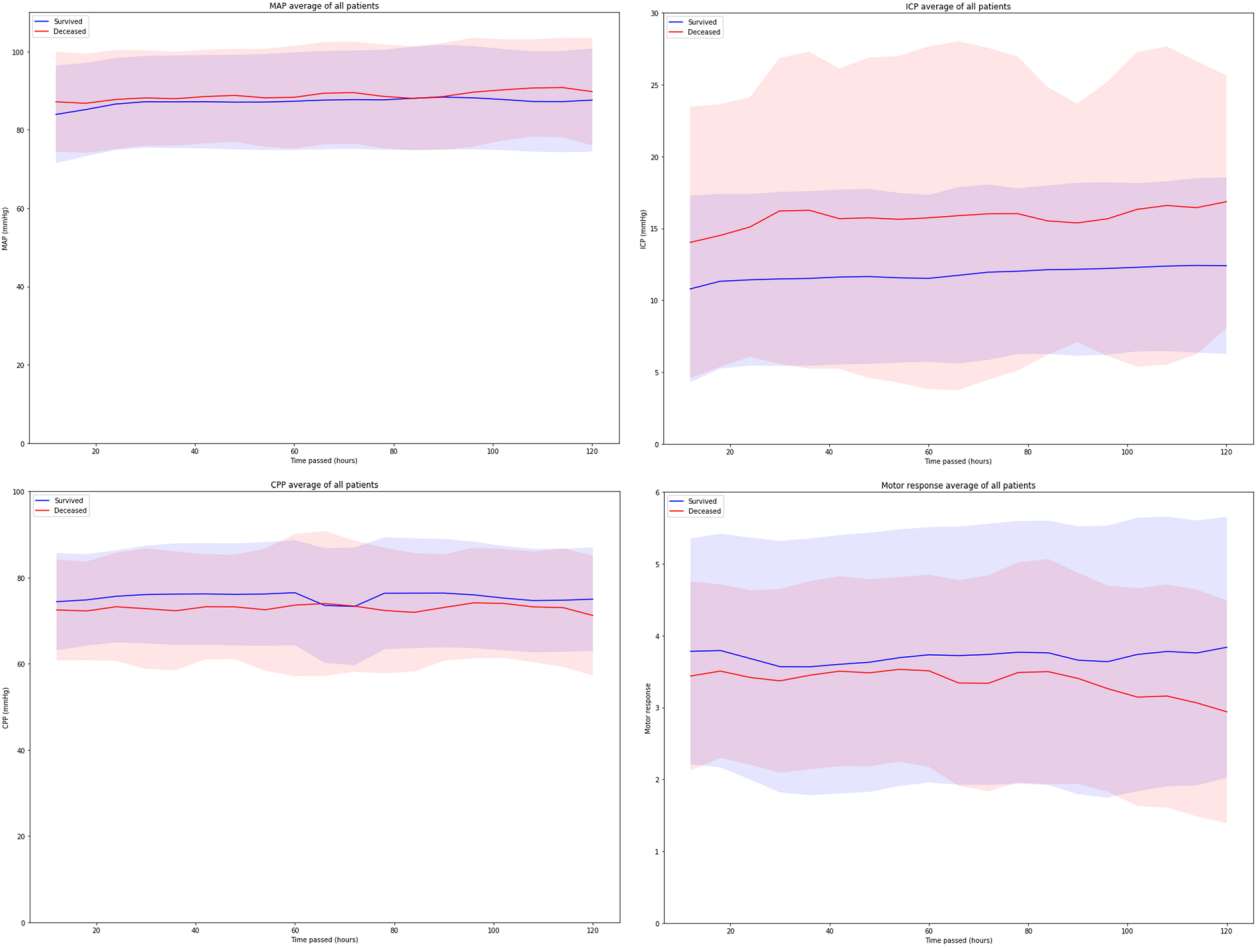
Up to the left mean arterial pressure (MAP), up to the right mean intracranial pressure (ICP), down to the left mean cerebral perfusion pressure (CPP) and down to the right mean motor response (MR) between survivors and survivors during the first 5 days. Means calculated in 12 h windows. Shown with 1 standard deviation. There was no notable difference in MAP between survivors and non-survivors. Mean ICP was higher and CPP was lower in non-survivors throughout. MR was lower for non-survivors and dropped towards the end.

The median age was 48 years, 69% of the patients had an admission GCS score of 3–8, 79% had two light reactive pupils, and 49% displayed a large mass lesion on the initial head CT. Overall 30-day mortality was 19% (N = 92). Differences in baseline characteristics between survivors and non-survivors are displayed in Table 1 and differences in baseline characteristics between the study centers are displayed in Table 1 in the Electronic Supplementary Material. Regarding the IMPACT-TBI variables, survivors were younger (p < 0.001), had higher median pre-intubation GCS scores (p = 0.009), more often two responsive pupils (p = 0.015), less often a mass lesion larger than 25 cm^3^ on the initial head CT (p = 0.019), lower admission blood glucose concentrations (p = 0.002), and higher admission hemoglobin concentrations (p = 0.043). There were no differences regarding pre-hospital hypoxia or hypotension between survivors and non-survivors (p = 0.38 and p = 0.67, respectively). There were no differences in rate of primary or secondary DCs between survivors and non-survivors (p = 0.13), and the mean ICP monitoring time between survivors and non-survivors (91 h vs. 85 h) was similar. Non-survivors had higher mean ICP, lower mean CPP, lower mean motor score, but similar mean MAP as survivors (Fig. 2).

**Table 1:** Patient baseline characteristics by survival status.

Variables	All patients (N = 472)	Survivors (N = 380)	Non-survivors (N = 92)	p-Value¶
**Age**	48 (32, 60)	45 (29, 58)	57 (44, 65)	<0.001
**Male**	379 (80%)	308 (81%)	71 (77%)	0.401
**Admission GCS score, median (IQR)**	6 (3, 10)	7 (4, 10)	6 (3, 8)	0.009
3–8	325 (69%)	253 (67%)	72 (79%)	0.094
9–12	108 (23%)	93 (24%)	15 (16%)	
13–15	39 (8%)	34 (9%)	5 (5%)	
**Motor score, median (IQR)**	4 (1, 5)	4 (2, 5)	4 (1, 5)	0.005
None/extension	148 (31%)	108 (28%)	40 (44%)	0.032
Abnormal flexion	28 (6%)	22 (6%)	6 (7%)	
Normal flexion	83 (18%)	68 (18%)	15 (16%)	
Localizes/obeys	213 (45%)	182 (48%)	31 (33%)	
**Pupillary light reactivity**				
Both react	373 (79%)	307 (81%)	66 (72%)	0.015
One reacts	75 (16%)	59 (15%)	16 (17%)	
None react	24 (5%)	14 (4%)	10 (11%)	
**Hypoxia**	78 (17%)	60 (16%)	18 (20%)	0.382
**Hypotension**	52 (11%)	43 (11%)	9 (10%)	0.673
**Marshall CT**				
DI I	9 (2%)	9 (2%)	0 (0%)	0.019
DI II	129 (27%)	115 (30%)	14 (15%)	
DI III	85 (18%)	64 (17%)	21 (23%)	
DI IV	20 (4%)	16 (4%)	4 (4%)	
EML/NEML	229 (49%)	176 (47%)	54 (58%)	
**tSAH on CT**	340 (72%)	267 (70%)	73 (79%)	0.082
**Epidural mass on CT**	46 (10%)	41 (11%)	5 (5%)	0.120
**Glucose** (mmol/l)*, median (IQR)	7.6 (6.6, 9,1)	7.5 (6.6, 9.8)	8.7 (6.9, 10.1)	0.002
**Hb** (g/l)†, median (IQR)	130 (118, 142)	131 (119, 143)	127 (113, 138)	0.043
**ICU length of stay**, median (IQR)	8 (4, 13)	9 (4, 14)	5 (3, 8)	<0.001
**Neurosurgical procedures**				
Craniotomy for mass lesion	224 (48%)	178 (47%)	46 (50%)	0.586
DC, total	73 (16%)	63 (17%)	10 (11%)	0.174
Primary DC	28 (6%)	22 (6%)	6 (7%)	0.130
Secondary DC	45 (10%)	41 (11%)	4 (4%)	
EVD	97 (21%)	77 (20%)	20 (22%)	0.753
**Mean predicted 30-day mortality‡** (95% CI)	20% (19, 20)	17% (16, 19)	32% (29, 35)	<0.001

Data shown as median with interquartile ranges and absolute numbers with percentages unless other specified.

*5 missing values.

†2 missing values.

‡Calculated using the IMPACT-TBI lab model for 467 patients and IMPACT-TBI extended model for 5 patients with missing glucose and Hb.

¶Between survivors and non-survivors.

Hypoxia is defined as a documented pre-hospital oxygen saturation of < 90% and hypotension is defined as a documented pre-hospital systolic blood pressure < 90 mmHg.

Abbreviations: EVD, External Ventricular Drain; DI, Diffuse Injury; DC, Decompressive Craniectomy; GCS, Glasgow Coma Scale; CT, Computerized Tomography; EML, Evacuated Mass Lesion larger than 25 cm^3^ NEML, Non-Evacuated Mass Lesion larger than 25 cm^3^ Hb, tSAH, traumatic subarachnoid hemorrhage; Hemoglobin; ICU, Intensive Care Unit.

### The ICP-MAP-CPP algorithm

**Figure 3:**
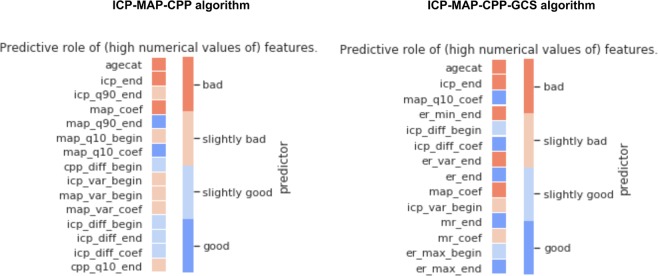
Heat map showing the predictive role of included features in the ICP-MAP-CPP algorithm (left) and the ICP-MAP-CPP-GCS algorithm (right). Red indicated that a higher feature value increases probability of 30-day mortality and blue indicates that a higher feature value increases probability of 30-day survival. Abbreviations: begin = mean value from the first derived 24-hour time-window; end = mean value from the last derived 8 hours; coef = slope of the linear coefficient from the start of the derived time-window up to the time of the prediction; q90 = 90th percentile in the derived time-window; q10 = 10th percentile in the derived time-window; diff = mean of differences between consequent values in the derived time-window; var = variance in the derived time-window; icp = intracranial pressure; cpp = cerebral perfusion pressure; map = mean arterial pressure; agecat = age category. See Table 2 for feature full feature description.

**Figure 4:**
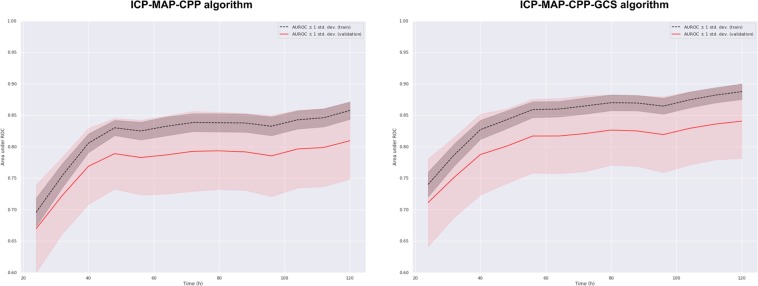
The algorithms’ AUC as a function of time by a repeated stratified cross validation technique. *Left*: the internal validation of the ICP-MAP-CPP algorithm showed an AUC of 0.67 on day 1, increasing to 0.81 on day 5. *Right:* The internal validation of the ICP-MAP-CPP-GCS algorithm showed an AUC of 0.72 on day 1, increasing to 0.84 by day 5.

**Figure 5:**
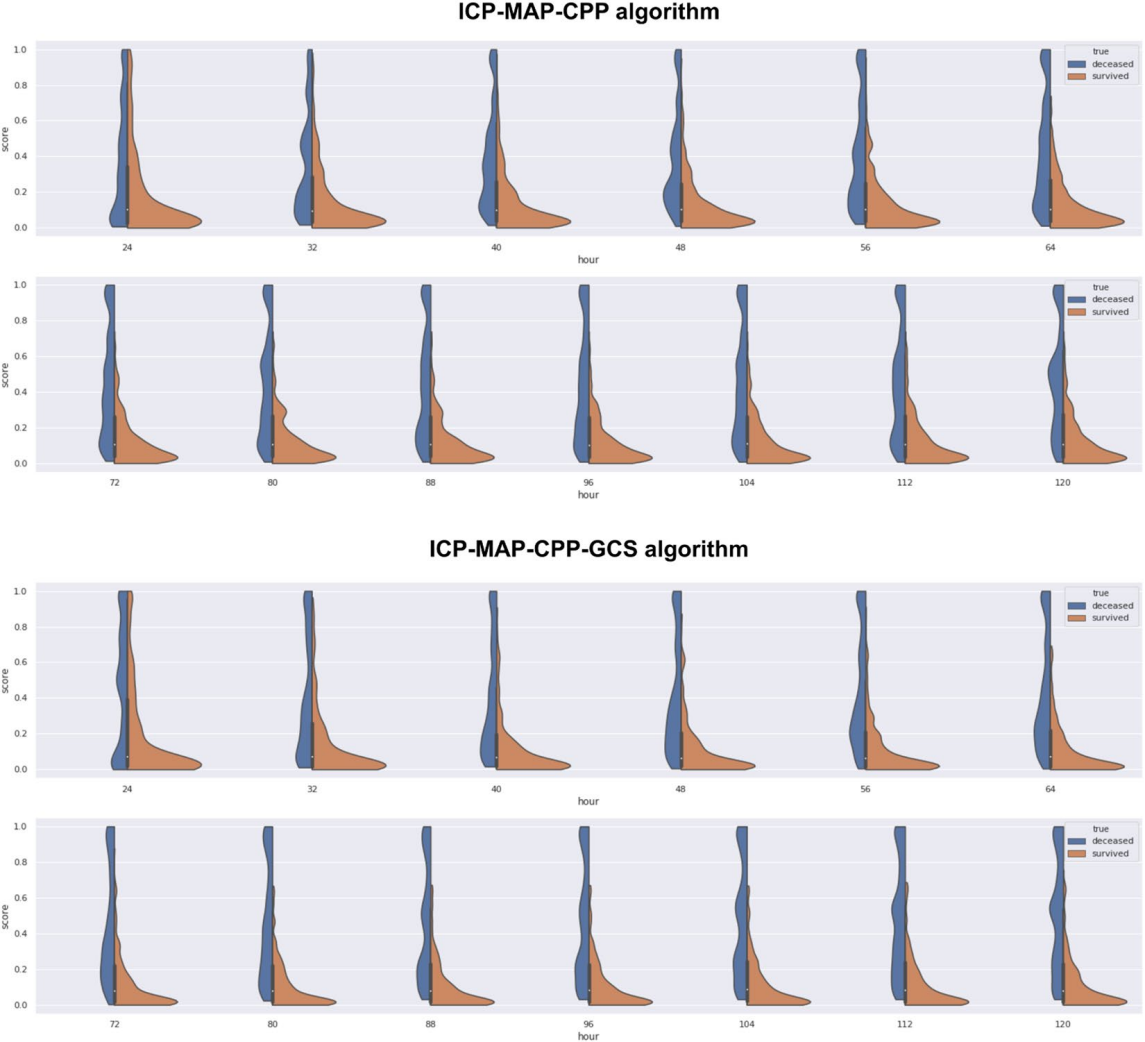
Violin plot showing the spectrum of predicted risks with time for survivors (orange) and non-survivors (blue). Upper image shows the ICP-MAP-CPP algorithm and the lower image show the ICP-MAP-CPP-GCS algorithm. Each figure goes from the first prediction (24 h) towards the last (120 h). For both algorithms the predicted risk for death increases with time for non-survivors (blue part gets thicker at the top and thinner at the bottom) and the predicted risk for death decreases with time for survivors (orange part gets thicker at the bottom and thinner at the top).

**Figure 6:**
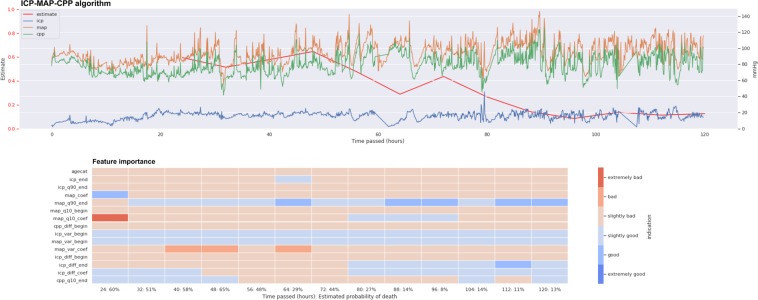
Example showing the ICP-MAP-CPP algorithm for a patient with a non-fatal 30-day outcome. The upper part shows the prediction (red line, higher value for higher probability of death) and the trends of intracranial pressure (ICP, blue line), mean arterial pressure (MAP, orange line), cerebral perfusion pressure (CPP, green line), eye response (ER, black dots) and motor response (MR, purple crosses). The lower part shows feature importance with time (red color indicating that a high feature value increases probability of death and blue color indicating that a high value increase probability of survival). The patient’s predicted risk of 30-day mortality according to the IMPACT-TBI based model was 70%.

Totally 14 dynamic features and patient age were included in the ICP-MAP-CPP algorithm (Table 2). The GCP code for the ICP-MAP-CPP algorithm is shown in Note 3 in the Electronic Supplementary Material. The predictive role of the included features is shown in Fig. 3 and the regression coefficients are showed in Table 2 in the Electronic Supplementary Material. The algorithm’s AUC increased from 0.67 (95% CI 0.60–0.74) on day one to 0.81 (95% CI 0.75–0.87) on day five (Fig. 4). A violin plot shows how the algorithm’s survival predictions function better in a time-dependent manner (Fig. 5). An example of dynamic predictions for one survivor is shown in Fig. 6.

**Table 2:** Included features in relative importance order

Algorithm	Abbreviation	Feature description
ICP-MAP-CPP	icp_end	mean ICP value in the last 8 h
	agecat	age category
	map_q10_coef	linear coefficient of MAP’s 10^th^ percentile in last time-window
	cpp_diff_begin	mean of differences in CPP in the first 24 h
	map_q90_end	mean value of MAP’s 90^th^ percentile in the last 8 h
	map_coef	linear coefficient of MAP
	icp_q90_end	mean value of ICP’s 90^th^ percentile in the last 8 h
	icp_diff_end	mean of differences in ICP in the last 8 h
	icp_diff_coef	linear coefficient of the mean of differences in ICP in the last time-window
	map_var_begin	variance of MAP in the first 24 h
	icp_var_begin	variance of ICP in the first 24 h
	icp_diff_begin	mean of differences in ICP in the first 24 h
	cpp_q10_end	mean value of CPP’s 10^th^ percentile in the last 8 h
	map_var_coef	linear coefficient of MAP’s variance in the last time-window
	map_q10_begin	mean value of MAP’s 10^th^ percentile in the first 24 h
ICP-MAP-CPP-GCS	er_min_end	worst eye response in the last 8 h
	icp_end	mean ICP value in the last 8 h
	er_max_end	best eye response in the last 8 h
	agecat	age category
	map_q10_coef	linear coefficient of MAP’s 10^th^ percentile in last time-window
	er_end	mean eye response value in the last 8 h
	map_coef	linear coefficient of MAP
	mr_end	mean motor response in the last 8 h
	icp_diff_begin	mean of differences in ICP in the first 24 h
	icp_diff_coef	linear coefficient of the mean of differences in ICP in the last time-window
	icp_var_begin	variance of ICP in the first 24 h
	er_var_end	variance of eye response in the last 8 h
	mr_coef	linear coefficient of motor response
	er_max_begin	best eye response in the first 24 h

Abbreviations: icp, intracranial pressure; cpp, cerebral perfusion pressure; map, mean arterial pressure; mr, motor response; er, eye response. For full feature abbreviation list and relative importance measures please see Note 2 in the Electronic Supplementary Material.

The number of false positives was 18. Of these, 7 were monitored only for 24 h to 48 h, 7 had a last prediction just slightly over 50% (50–57%), and 4 had a last prediction of 62–74%. Of these four patients (prediction 62 to 74%), three recovered to live dependently in a nursing home and one was left severely disabled and bedridden (Fig. 1 in the Electronic Supplementary Material). Furthermore, three out of these four patients underwent decompressive craniectomy, suggesting that our algorithm, which includes ICP, may not be reliable in these patients.

### The ICP-MAP-CPP-GCS algorithm

**Figure 7:**
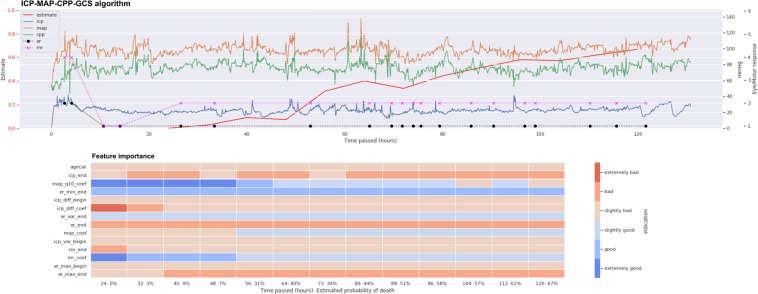
Example showing the ICP-MAP-CPP-GCS algorithm for a patient with a fatal 30-day outcome. The upper part shows the prediction (red line, higher value for higher probability of death) and the trends of intracranial pressure (ICP, blue line), mean arterial pressure (MAP, orange line), cerebral perfusion pressure (CPP, green line), eye response (ER, black dots) and motor response (MR, purple crosses). The lower part shows feature importance with time (red color indicating that a high feature value increases probability of death and blue color indicating that a high value increase probability of survival). The patient’s predicted risk of 30-day mortality according to the IMPACT-TBI based model was 42%.

Totally 13 dynamic features and patient age were included in the final ICP-MAP-CPP-GCS algorithm (Table 2). The GCP code for the ICP-MAP-CPP-GCS algorithm is shown in Note 4 in the Electronic Supplementary Material (see Table 2 in the Electronic Supplementary Material for regression coefficients). Heat maps show the temporal profile of feature importance during the monitoring period (Fig. 3). The algorithm’s AUC following cross validation was slightly higher than that of the ICP-MAP-CPP algorithm and increased from 0.72 (95% CI 0.64–0.78) to 0.84 (95% CI 0.78–0.90) from day one to day five (Fig. 4). The violin plot for the ICP-MAP-CPP-GCS algorithm is shown in Fig. 5. An example of dynamic predictions for one non-survivor is shown in Fig. 7.

### IMPACT-TBI based model

**Figure 8:**
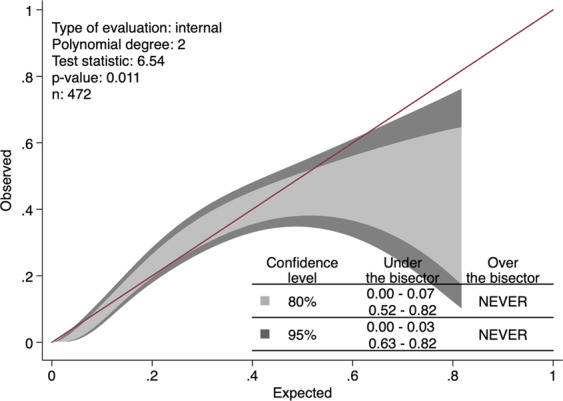
Calibration belt for the IMPACT-TBI model based on admission characteristics showing that the model overestimates the risk of death for patients with an initial prognosis of 0–3% and 63–82% (95% confidence intervals).

According to the IMPACT-TBI model, non-survivors had a significantly higher mean predicted risk of 30-day mortality than survivors (mean 32% vs. 17%, p < 0.001). The AUC of the IMPACT-TBI model for predicting 30-day mortality was 0.78 (95% CI 0.73, 0.82) and the calibration belt shows that the IMPACT-TBI model overestimated the risk of death for patients with an initial very good or poor prognosis (Fig. 8). Using a cut-off of 50% for predicting death, the IMPACT-TBI model estimated 30 patients to die, out of which 15 patients (50%) died within 30-days and 15 patients (50%) survived (these survivors represent false positives). For these same 30 patients, the ICP-MAP-CPP algorithm misclassified 11 patients (2 false positives and 9 false negatives) and the ICP-MAP-CPP-GCS algorithm misclassified 5 patients (1 false positive and 4 false negatives).

## Discussion

We demonstrated that it is possible to capture patient-level dynamic changes in prognosis during intensive care after TBI by the novel machine learning-based algorithms. To date this has not been made, as current prognostic models for TBI are based upon static admission characteristics^9,10,24^. Given that our aim was to create dynamic but simple algorithms that could be widely used in ICUs worldwide, our algorithms consisting of only three to four main variables, seemed to fulfil the objective as the algorithms’ performance was good and the risk of false-positive predictions was low. Furthermore, our concept of using low-frequency (5-minute medians) big data overcomes the major challenges for practical use of high-frequency big data in clinical settings. In other words, the algorithms work even in conditions when monitoring is intermittently disrupted, as happens often in ICUs. In brief, we believe that the presented algorithms are a promising start in developing novel big data algorithms predicting TBI outcome. Such algorithms hold the potential in aiding clinicians to make more timely, data-driven and standardized treatment decisions.

Of the two algorithms, the ICP-MAP-CPP algorithm was completely automatized and relied only on monitor data, giving no room for subjective interpretations of patient’s clinical condition. In contrast, the ICP-MAP-CPP-GCS algorithm is subject to human-error and can be argued to be less reliable and generalizable (especially in centers without neurointensive care units), although the addition of the GCS components increased the algorithm’s discrimination. We argue that the algorithms could be further improved with larger datasets in order to allow more powerful and sophisticated machine learning approaches. Therefore, hopefully the presented results will be of interest, and we could further develop the algorithms through a multinational collaboration.

### Algorithm performance

The concept of assessing performance for dynamic prognostic models is rather unexplored. Traditionally, the performance of static prognostic models are tested by assessing discrimination (i.e. ability to distinguish between those with and without the defined outcome, AUC for binary outcome models) and calibration (i.e. the agreement of observed outcomes with predicted risk, usually by Hosmer-Lemeshow goodness-of-fit test and/or calibration plots)^25^. However, for dynamic prediction algorithms, the predictions change in a time-dependent manner and thus, assessing AUC and calibration at one arbitrary time point would be misleading. For example, if we had reported only the best AUC values in any given time point, the results would probably have been overoptimistic. Thus, we tackled the discrimination problem by calculating a time-dependent AUC curve (with a repeated stratified cross validation technique) to show the algorithms’ discriminative performance over time. The ICP-MAP-CPP algorithm predicted 30-day mortality with a discrimination of 67% on day one to 81% on day five (new predictions given in 8 h intervals after the first 24 h). The ICP-MAP-CPP-GCS algorithm predicted 30-day mortality with a discrimination of 72% on day one to 84% on day five. Still, notable is that both algorithms reached their maximum AUC after approximately 48 hours, after which the increase in AUC was less noticeable. It is likely that by increasing algorithm complexity and by adding a higher number of features the AUC could have been further increased, however, with the cost of overfitting and reduced generalizability. This is well in line with the basic concepts of big data analytics, which suggests that with a larger cohort size and more variables, the results could improve.

Regarding calibration, there are no standards how to assess it for dynamic prediction models. We show a version of the algorithms’ calibration by violin plots. The violin plots show that the number of correctly predicted patients increases with time, which suggests that the calibration improves with increasing time. Regarding the IMPACT-TBI-based model’s calibration, we found that the model overestimated the risk of 30-day mortality for those with a very low probability of death and for those with a very high probability of death. Such overestimation (i.e. false positives) should always be dealt with caution, as a poor prognostic estimate becomes easily a self-fulfilling prophecy in critically ill patients. Using a pre-defined arbitrary cut-off of 50% for the IMPACT-TBI model, half of the patients survived although their initial IMPACT-TBI prognosis was higher than 50% (i.e. false positives). In contrast, only one in five patients survived if the dynamic algorithm’s last prediction was higher than 50%, indicating that the calibration for the dynamic algorithm outperforms the static model, especially for high-risk patients. Yet, we highlight that the IMPACT-TBI model was not developed for prognostication at the individual level, but rather to facilitate TBI research by improving study design and enabling case-mix adjustment^26^. Thus, the introduction of dynamic prognostic algorithms does not mean that we should abandon the static models, as they are intended for different purposes. So, although the difference in AUC between the static and dynamic models was rather similar (0.78 for the static model compared to a maximum of 0.84 for the ICP-MAP-CPP-GCS model on day five), their purposes are widely different. The real-time predictions that are based on the dynamic algorithms could be used to alert the physician about subtle neuroworsening and to quantify the effect of different medical and surgical interventions on prognosis. With larger sample sizes and more robust machine learning techniques it is possible to further develop the models to suggest the most optimal course of treatment to optimize patient outcome^27^.

It is important to notice that we decreased the likelihood of overfitting by excluding patients dying within 36 h and by excluding the last 12 h of monitor data for those dying within the first five days. Including early fatal outcomes, that represent the extreme events of abnormal intracranial physiology, would have made it substantially easier to discriminate between survivors and non-survivors, but with the cost of overfitting and compromised generalizability. It is worth mentioning that the decision to give the first prediction after 24 h and the subsequent predictions in 8 h intervals was arbitrary and it is possible to change the length of these intervals in the provided code. Whether it is relevant to frequently gain new predictions is something that still needs exploring.

Previously, even the benefits of measuring ICP in TBI patients at all has been questioned^28^. The presented algorithms include ICP features as some of their most important components. Of the 14 included dynamic features in the ICP-CPP-MAP algorithm, six were ICP-derived features and 2 were CPP-derived. The most significant ICP features were the mean ICP of the last 8 h, the 90^th^ percentile of ICP, the mean of differences between consequent values in the last time window, and the linear coefficient of the mean difference between consequent values in the last time window. Such dynamic features are complex and virtually impossible for a clinician to take into account on daily decision-making processes in the ICU, but still comprehensible when computed by a machine learning approach. Moreover, six features in the ICP-CPP-MAP algorithm, were based upon MAP. In comparison, in the IMPACT-TBI model, the hemodynamic status is represented by a single binary variable of hypotension and does not account for insult duration. According to our results, also later hemodynamic features and trends seem to contribute to patient prognosis. Furthermore, the prognostic importance of the GCS is highlighted once again, as 7 out of 13 dynamic features in the ICP-MAP-CPP-GCS algorithm were derived from either the motor or eye response. Interestingly, features based on eye response were of higher importance than features based on motor response. In contrast, when prognostic factors are measured upon admission, motor response has been shown to be the more important predictor^29^. Worth mentioning is also that five features of the ICP-CPP-MAP algorithm and three features of the ICP-CPP-MAP-GCS algorithm were “_begin” features (accounting for the first 24 h), highlighting the importance of the first 24 hours.

Our 30-day mortality was rather low (19%) compared to previous series showing mortality rates up to 40–50%^30–32^. This is related to the fact that approximately one-third of the patients had an admission GCS of 9 or more (although these were ICP monitored). Still, 30-day mortality for those with an admission GCS of 3–8 was only 22% (compared to 14% and 13% for those with an admission GCS of 9–12 and 13–15, respectively). As mentioned before, it is important to notice that we did not include patients who were ICP monitored less than 24 h or who died within 36 h of admission, causing the mortality numbers to be lower than they really are. This was a deliberate decision, as we wanted to avoid withdrawal of treatment to affect the algorithms’ predictions. Further, one could argue that the additional value of prognostic algorithms in the assessment of patients with so severe TBIs, dying within 36 h, is limited.

### Previous models

There are no previous dynamic outcome prediction models for ICU-treated TBI patients. Güiza and colleagues used a cohort of 264 patients and added dynamic ICP features from the first 24 h to the static IMPACT-TBI and CRASH models, and demonstrated an increase in AUC from 0.68–0.72 to 0.87–0.90^33^. Although the model is not purely static and includes dynamic features of ICP, it gives one prediction after 24 h and is thus, similar to the Acute Physiology and Chronic Health Evaluation (APACHE) and Simplified Acute Physiology Score (SAPS) scoring systems used for the general intensive care population^34–36^. Bonds and colleagues used a nearest neighbor regression technique to predict future ICP fluctuations using vital parameters such as ICP, heart rate, systolic blood pressure, shock index, MAP and pulse pressure^37^. Also, Donald and colleagues used Bayesian artificial neural networks to dynamically predict hypotensive insults during neurocritical care using a total of 15 features of MAP, systolic blood pressure, heart rate plus age and sex and demonstrated an AUC between 0.65–0.74^38^. Rau and colleagues used several static admission characteristics and compared different machine learning algorithms and found that artificial neural network and logistic regression showed superior performance for predicting hospital mortality compared to other machine learning algortihms^39^.

### Strengths and limitations

There are some strengths and limitations that should be declared. First, although a machine-learning logistic regression technique demonstrated the most reliable and consistent results, it is possible that more advanced machine learning techniques than logistic regression could increase algorithm performance. However, logistic regression has been shown to outperform more sophisticated machine learning techniques in terms of TBI mortality prediction based upon static variables^40^. Moreover, by using a logistic regression approach, we were able to assess which variables contribute to each prediction. We believe that this approach is advantageous in early steps of building new clinical algorithms, as it gives us a better understanding of how such algorithms work and which variables are truly valuable for dynamic outcome prediction, with the cost of capturing complex inter-feature relationships. For example, unsupervised machine learning approaches are more or less so-called “black box” approaches, leaving many aspects of the algorithm ambiguous, and the study results would be challenging to repeat in other centers. Still, by using more sophisticated techniques, such as artificial neural networks or clustering, it is possible to design more powerful algorithms including more sophisticated features than what is possible with logistic regression.

Second, we used 30-day mortality as our outcome measure because it is definitive and most likely related to TBI-death. However, using a longer follow-up would probably have increased the AUC with the cost of including deaths due to other causes than TBI^41^. Furthermore, as shown by Adams and colleagues, ICP correlates poorly with functional outcome, limiting dynamic predictions of functional outcome measures^41^.

Third, we tried to calibrate the algorithms to minimize the number of false positives (i.e. algorithm predicts death although the patient survives), since in a worst-case scenario, false positive predictions could become self-fulfilling prophecies leading to a detrimental withdrawal of treatment. However, we were still left with some false positives. After reviewing the false positives in a post-hoc analysis, we believe that only 4 out of 18 were true false positives (Fig. 1 in the Electronic Supplementary Material). Three out of these underwent decompressive craniectomy, meaning that increases in ICP are effectively repressed, making it difficult for an ICP-based algorithm to predict the outcome. One should also remember that the definition of a false positive for a dynamic algorithm is not clear (we used the last given prediction), and that the purpose of such algorithm is not to work alone but to summarize vast amount of information for the clinician in an easily interpretable manner. It is plausible that by integrating automatized head CT image analysis into the risk estimation, the number of false positives would decrease.

Fourth, included monitor data (ICP, MAP, CPP) are all subject to manipulation by treatment. For example, a patient with an ICP level of 15–20 mmHg requiring no ICP lowering treatment and a patient with a similar ICP level requiring rigorous ICP lowering interventions are probably not the same in terms of prognosis. However, the algorithms were built to ignore such vastly diverse data. This considered, we think that the algorithms worked exceptionally well, and the results are quite promising. In the future, however, larger sample sizes may allow for incorporation of given treatments and treatment targets, which may improve prognostic performance and also may enable data-driven personalized treatment plans.

Fifth, we chose not to imputate missing values as we believe this mimic the real clinical situation where values are normally missing during different aspects of care (e.g. catheter flushing, patient transfers). Still, this may include a risk of bias.

Sixth, our algorithms do not provide estimates of errors for the individual predictions, which is a limitation. With further developments of dynamic prediction models, it is important to include error estimations.

Seventh, the presented algorithms are based on a patient cohort of three academic centers from one country, and the algorithms require further multinational external validation.

## Conclusion

We present the first dynamic prognostic algorithms for real-time outcome prediction of patients with TBI treated in the ICU. These simple algorithms are based on three to four main variables and are, in contrast to current static prognostic models, dynamic in nature and may aid in clinical decision making. The concept of using low-frequency big data for clinically applicable algorithms seems promising. The dynamic algorithms are open-sourced and free to be used for further development, also in the LMIC setting. With additional multicenter studies, also LMIC centers included, these predictive algorithms are likely to be improved. We believe, that an internationally validated algorithm that could capture dynamic changes in prognosis during intensive care could aid clinicians to make more data-driven treatment decisions, potentially improving quality of care.

## Supplementary information

Supplementary Information

## Data Availability

According to national legislation, individual patient data is not freely shared.
