# Supplementary material for: Machine learning-based dynamic mortality prediction after traumatic brain injury
Source: Sci Rep. 2019 Nov 27;9:17672. doi: 10.1038/s41598-019-53889-6 (PMC6881446; doi:10.1038/s41598-019-53889-6)
Supplement: Supplementary file 1 — Supplementary Information [file 41598_2019_53889_MOESM1_ESM.pdf]

ELECTRONIC SUPPLEMENTARY MATERIAL

Title:
Machine learning-based dynamic mortality prediction after traumatic brain injury

Authors:
Rahul Raj, Teemu Luostarinen, Eetu Pursiainen, Jussi P Posti, Riikka SK Takala, Stepani Bendel, Teijo Konttila, Miikka Korja

Supplementary Content:

Note 1	Page 2
Note 2	Page 3
Figure 1	Page 8
Table 1	Page 9
Table 2	Page 10
Note 3	Page 11
Note 4	Page 26

Note 1

TRIPOD Checklist: Prediction Model Development

Section/Topic		Checklist Item	
**Title and abstract**			
Title	1	Identify the study as developing and/or validating a multivariable prediction model, the target population, and the outcome to be predicted.	OK
Abstract	2	Provide a summary of objectives, study design, setting, participants, sample size, predictors, outcome, statistical analysis, results, and conclusions.	OK
**Introductio**			
Background and objectives	3a	Explain the medical context (including whether diagnostic or prognostic) and rationale for developing or validating the multivariable prediction model, including references to existing models.	OK
	3b	Specify the objectives, including whether the study describes the development or validation of the model or both.	OK
**Methods**			
Source of data	4a	Describe the study design or source of data (e.g., randomized trial, cohort, or registry data), separately for the development and validation data sets, if applicable.	OK
	4b	Specify the key study dates, including start of accrual; end of accrual; and, if applicable, end of follow-up.	OK
Participants	5a	Specify key elements of the study setting (e.g., primary care, secondary care, general population) including number and location of centres.	OK
	5b	Describe eligibility criteria for participants.	OK
	5c	Give details of treatments received, if relevant.	OK
Outcome	6a	Clearly define the outcome that is predicted by the prediction model, including how and when assessed.	OK
	6b	Report any actions to blind assessment of the outcome to be predicted.	NA
Predictors	7a	Clearly define all predictors used in developing or validating the multivariable prediction model, including how and when they were measured.	OK
	7b	Report any actions to blind assessment of predictors for the outcome and other predictors.	NA
Sample size	8	Explain how the study size was arrived at.	OK
Missing data	9	Describe how missing data were handled (e.g., complete-case analysis, single imputation, multiple imputation) with details of any imputation method.	OK
Statistical analysis methods	10a	Describe how predictors were handled in the analyses.	OK
	10b	Specify type of model, all model-building procedures (including any predictor selection), and method for internal validation.	OK
	10d	Specify all measures used to assess model performance and, if relevant, to compare multiple models.	OK
Risk groups	11	Provide details on how risk groups were created, if done.	NA
**Results**			
Participants	13a	Describe the flow of participants through the study, including the number of participants with and without the outcome and, if applicable, a summary of the follow-up time. A diagram may be helpful.	OK
	13b	Describe the characteristics of the participants (basic demographics, clinical features, available predictors), including the number of participants with missing data for predictors and outcome.	OK
Model development	14a	Specify the number of participants and outcome events in each analysis.	OK
	14b	If done, report the unadjusted association between each candidate predictor and outcome.	OK
Model specification	15a	Present the full prediction model to allow predictions for individuals (i.e., all regression coefficients, and model intercept or baseline survival at a given time point).	OK
	15b	Explain how to the use the prediction model.	OK
Model performance	16	Report performance measures (with CIs) for the prediction model.	OK
**Discussion**			
Limitations	18	Discuss any limitations of the study (such as nonrepresentative sample, few events per predictor, missing data).	OK
Interpretation	19b	Give an overall interpretation of the results, considering objectives, limitations, and results from similar studies, and other relevant evidence.	OK
Implications	20	Discuss the potential clinical use of the model and implications for future research.	OK
**Other information**			
Supplementary information	21	Provide information about the availability of supplementary resources, such as study protocol, Web calculator, and data sets.	OK
Funding	22	Give the source of funding and the role of the funders for the present study.	OK

Note 2

Features and feature elimination processes

Abbreviations:
agecat = age category (20, 20-29, 30-39, 40-49, 50-59, 60-69, 70-79, 80-89, 90-99 years)
icp = intracranial pressure
cpp = cerebral perfusion pressure
map = mean arterial pressure
mr = motor response
er = eye response

begin = mean value from the first derived 24-hour time-window
end = mean value from the last derived 8 hours
coef = slope of the linear coefficient from the start of the derived time-window up to the time of the prediction

min = minimum value in the derived time-window
max = maximum value in the derived time-window
diff = mean of differences between consequent values in the derived time-window
var = variance in the derived time-window
avg = mean value in the derived time-window

q90 = 90th percentile in the derived time-window
q10 = 10th percentile in the derived time-window
ht20 = percentage of measurement points being higher than 20 mmHg in the derived time-window
ht120 = percentage of measurement points being higher than 120 mmHg in the derived time-window
lt10 = percentage of measurement points being lower than 10 mmHg in the derived time-window

Since we used a stratified cross validation technique the included features might differ depending on how the folds are randomized.

The ICP-MAP-CPP algorithm

1. Total of 55 features considered:

- agecat
- icp_begin
- icp_end
- icp_coef
- icp_q90_begin
- icp_q90_end
- icp_q90_coef
- cpp_var_begin
- cpp_var_end
- cpp_var_coef
- map_begin
- map_end
- map_coef
- map_q90_begin
- map_q90_end
- map_q90_coef
- map_q10_begin
- map_q10_end
- map_q10_coef
- map_diff_begin
- map_diff_end
- map_diff_coef
- cpp_diff_begin
- cpp_diff_end
- cpp_diff_coef
- icp_ht20_begin
- icp_ht20_end
- icp_ht20_coef
- icp_var_begin
- icp_var_end
- icp_var_coef
- map_ht120_begin
- map_ht120_end
- map_ht120_coef
- map_var_begin
- map_var_end
- map_var_coef
- cpp_begin
- cpp_end
- cpp_coef
- icp_diff_begin
- icp_diff_end
- icp_diff_coef
- icp_lt10_begin
- icp_lt10_end
- icp_lt10_coef
- cpp_q10_begin
- cpp_q10_end
- cpp_q10_coef
- icp_q10_begin
- icp_q10_end
- icp_q10_coef
- cpp_q90_begin
- cpp_q90_end
- cpp_q90_coef

2. Recursive feature elimination: optimal number of features = 15

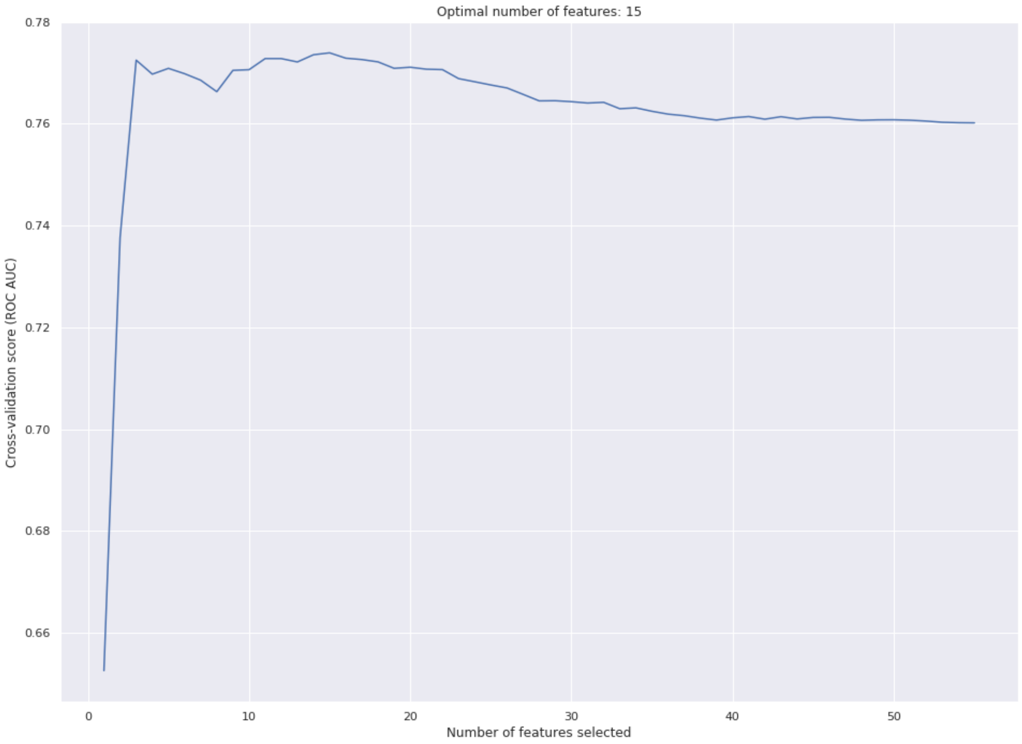

3. Relative feature importance

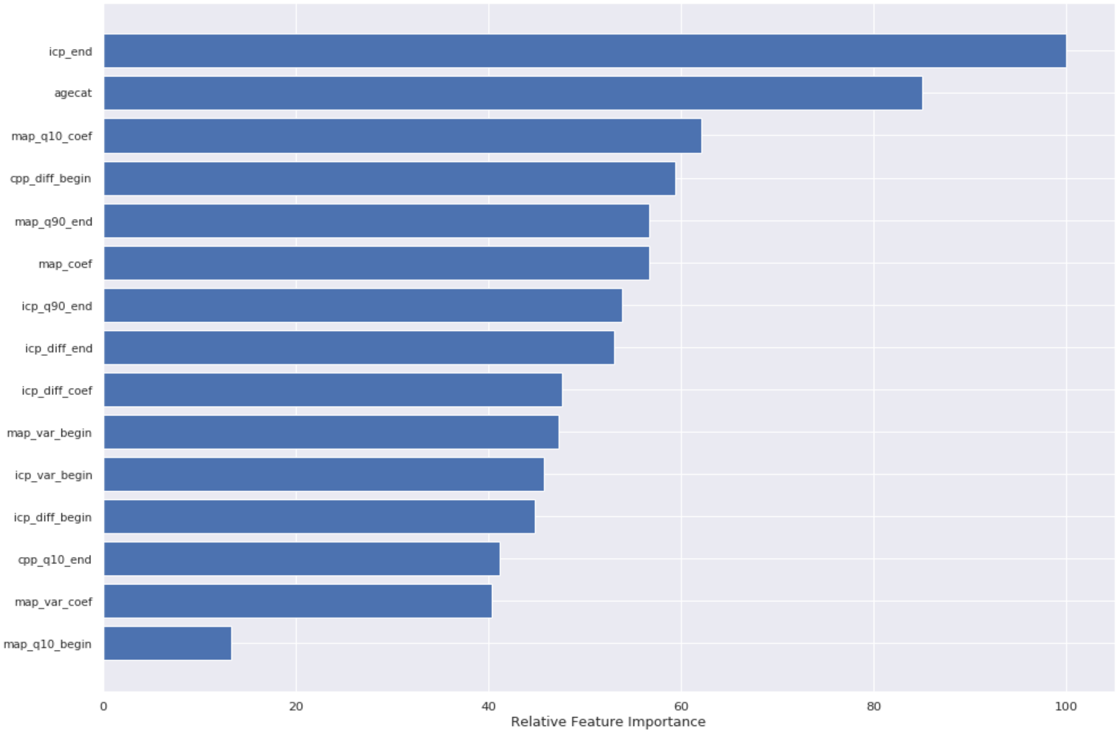

The ICP-MAP-CPP-GCS algorithm

1. Total of 76 features considered:

- agecat
- icp_begin
- icp_end
- icp_coef
- cpp_begin
- cpp_end
- cpp_coef
- map_q10_begin
- map_q10_end
- map_q10_coef
- cpp_q10_begin
- cpp_q10_end
- cpp_q10_coef
- icp_ht20_begin
- icp_ht20_end
- icp_ht20_coef
- er_min_begin
- er_min_end
- er_min_coef
- mr_var_begin
- mr_var_end
- mr_var_coef
- map_ht120_begin
- map_ht120_end
- map_ht120_coef
- er_avg_begin
- er_avg_end
- er_avg_coef
- cpp_diff_begin
- cpp_diff_end
- cpp_diff_coef
- cpp_q90_begin
- cpp_q90_end
- cpp_q90_coef
- icp_diff_begin
- icp_diff_end
- icp_diff_coef
- er_var_begin
- er_var_end
- er_var_coef
- icp_q90_begin
- icp_q90_end
- icp_q90_coef
- map_q90_begin
- map_q90_end
- map_q90_coef
- er_begin
- er_end
- er_coef
- cpp_var_begin
- cpp_var_end
- cpp_var_coef
- map_var_begin
- map_var_end
- map_var_coef
- map_begin
- map_end
- map_coef
- map_diff_begin
- map_diff_end
- map_diff_coef
- icp_q10_begin
- icp_q10_end
- icp_q10_coef
- icp_var_begin
- icp_var_end
- icp_var_coef
- mr_begin
- mr_end
- mr_coef
- icp_lt10_begin
- icp_lt10_end
- icp_lt10_coef
- er_max_begin
- er_max_end
- er_max_coef

2. Recursive feature elimination: optimal number of features = 14

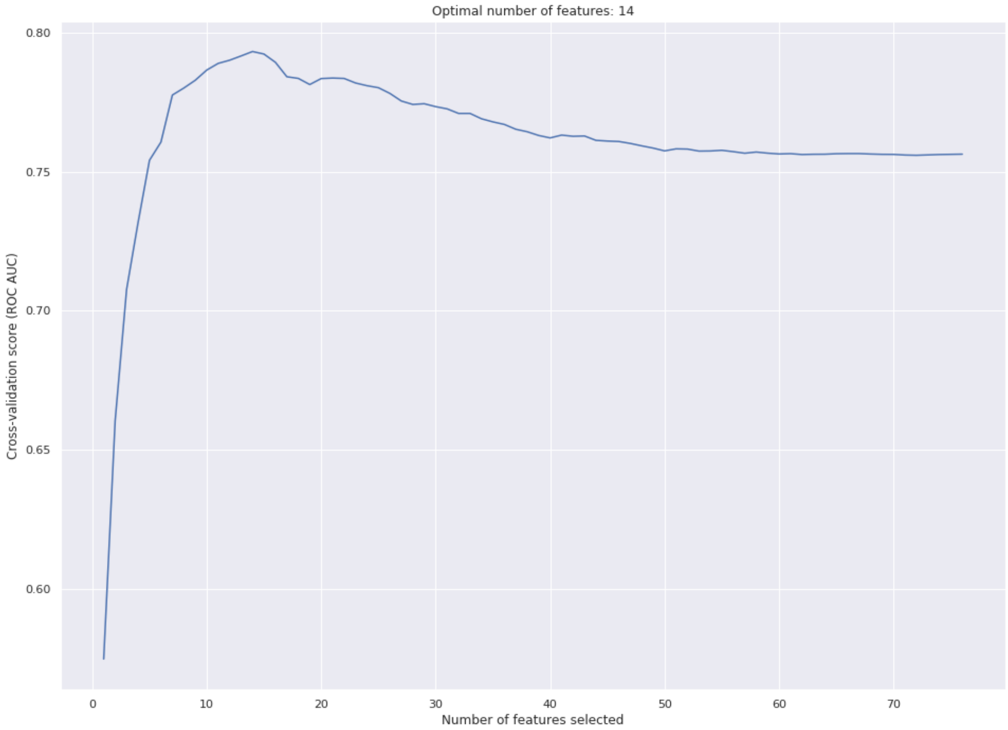

3. Relative feature importance

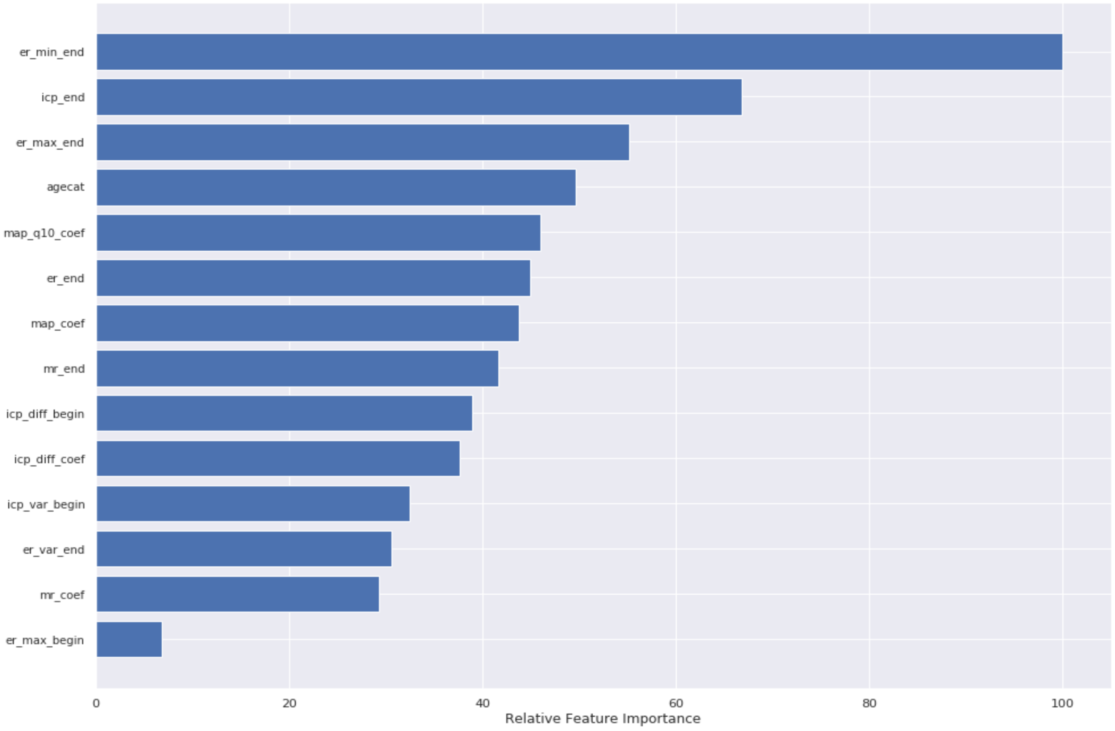

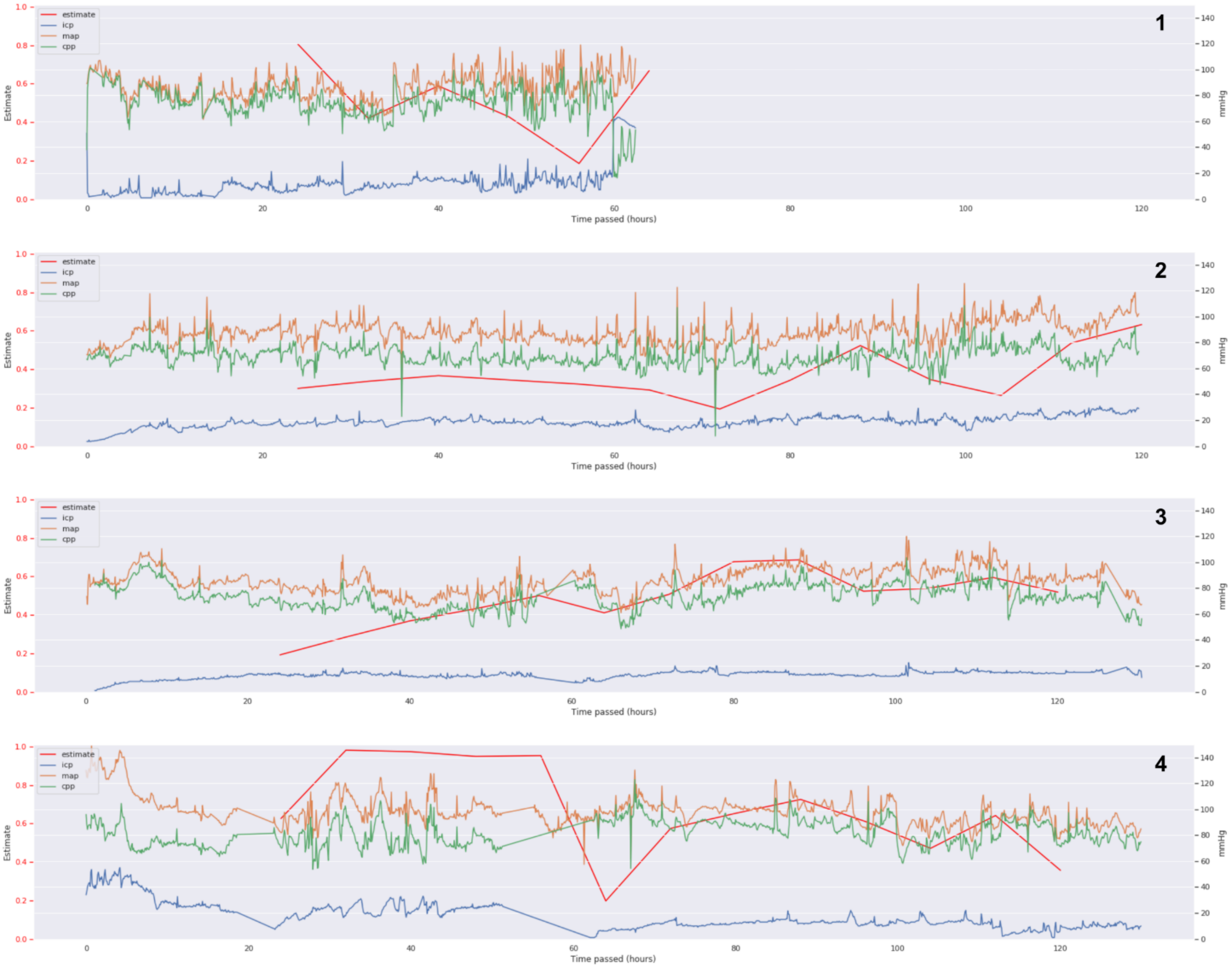

Supplementary Figure 1: Showing the predictions of the four false negatives with a last probability of death over 60%. All of these patients survived. Numbers one to three recovered to live dependently in a nursing home facility and number four was left severely disabled and bedridden. Numbers two to four underwent decompressive craniectomy that effectively lowered their rise in intracranial pressure.

Supplementary Table 1: Patient baseline characteristics by study center

Variables	HUS (N=185)	KUH (N=127)	TUH (N=160)	p-Value
**Age**	45 (42-63)	55 (42-63)	44 (27-61)	<0.001
**Male**	144 (78%)	103 (81%)	132 (83%)	0.56
**Admission GCS score**, median (IQR)	6 (3-8)	6 (4-9)	7 (5-11)	<0.001
3-8	140 (76%)	90 (71%)	95 (59%)	<0.001
9-12	42 (22%)	21 (17%)	45 (28%)	
13-15	3 (2%)	16 (13%)	20 (13%)	
**Motor score**, median (IQR)				
None/extension	74 (40%)	37 (29%)	37 (23%)	0.002
Abnormal flexion	7 (4%)	7 (6%)	14 (9%)	
Normal flexion	24 (13%)	32 (25%)	27 (17%)	
Localizes/obeys	80 (43%)	51 (40%)	82 (51%)	
**Pupillary light reactivity**				
Both react	134 (72%)	104 (82%)	135 (84%)	0.08
One reacts	38 (21%)	18 (14%)	19 (12%)	
None react	13 (7%)	5 (4%)	6 (4%)	
**Hypoxia**	35 (19%)	23 (18%)	20 (13%)	0.24
**Hypotension**	26 (14%)	12 (9%)	14 (9%)	0.24
**Marshall CT**				
DI I	0 (0%)	4 (3%)	5 (3%)	<0.001
DI II	40 (22%)	23 (18%)	66 (41%)	
DI III	36 (20%)	24 (19%)	25 (16%)	
DI IV	10 (5%)	4 (3%)	6 (4%)	
EML/NEML	99 (53%)	72 (57%)	58 (36%)	
**tSAH on CT**	158 (85%)	67 (53%)	115 (72%)	<0.001
**Epidural mass on CT**	18 (10%)	11 (9%)	17 (11%)	0.86
**Glucose** (mmol/l)*, median (IQR)	7.6 (6.5-9.2)	7.8 (6.9-9.1)	7.4 (6.6-9.1)	0.33
**Hb** (g/l)†, median (IQR)	131 (119-144)	129 (117-140)	132 (116-142)	0.32
**ICU length of stay**, median (IQR)	10 (5-14)	4 (2-8)	9 (5-17)	<0.001
**Neurosurgical procedures**				
Craniotomy for mass lesion	88 (48%)	80 (63%)	56 (35%)	<0.001
DC, total	32 (17%)	19 (15%)	22 (14%)	0.65
Primary DC	16 (50%)	8 (42%)	4 (18%)	0.06
Secondary DC	16 (50%)	11 (58%)	18 (82%)	
EVD	29 (16%)	57 (45%)	11 (7%)	<0.001
**Observed 30-day mortality**	26 (14%)	27 (21%)	39 (24%)	0.046S,
**Mean predicted 30-day mortality‡** (95% CI)	21% (18-23)	18% (15-20)	22% (19-25)	0.06

Data shown as median with interquartile ranges and absolute numbers with percentages unless other specified.

*5 missing values.
†2 missing values.
‡Calculated using the IMPACT-TBI lab model for 467 patients and IMPACT-TBI extended model for 5 patients with missing glucose and Hb.

Hypoxia is defined as a documented pre-hospital oxygen saturation of <90% and hypotension is defined as a documented pre-hospital systolic blood pressure <90 mmHg.

Abbreviations: HUS, Helsinki University Hospital; KUH, Kuopio University Hospital; TUH, Turku University Hospital; EVD, External Ventricular Drain; DI, Diffuse Injury; DC, Decompressive Craniectomy; GCS, Glasgow Coma Scale; CT, Computerized Tomography; EML, Evacuated Mass Lesion larger than 25 cm^3; NEML, Non-Evacuated Mass Lesion larger than 25 cm^3; Hb, tSAH, traumatic subarachnoid hemorrhage; Hemoglobin; ICU, Intensive Care Unit

Supplementary Table 2: Included features' regression coefficients

Algorithm	Abbreviation	Coefficient (SD)
**ICP-MAP-CPP**	icp_end	1.350 (0.329)
	agecat	0.998 (0.116)
	map_q10_coef	-0.817 (0.543)
	cpp_diff_begin	-0.764 (0.165)
	map_q90_end	-0.920 (0.187)
	map_coef	0.776 (0.484)
	icp_q90_end	0.679 (0.412)
	icp_diff_end	-0.554 (0.345)
	icp_diff_coef	-0.767 (0.312)
	map_var_begin	0.668 (0.170)
	icp_var_begin	0.441 (0.281)
	icp_diff_begin	-0.563 (0.125)
	cpp_q10_end	0.720 (0.179)
	map_var_coef	0.594 (0.245)
	map_q10_begin	0.155 (0.078)
**ICP-MAP-CPP-GCS**	er_min_end	2.076 (0.210)
	icp_end	1.205 (0.132)
	er_max_end	-1.072 (0.217)
	agecat	0.909 (0.101)
	map_q10_coef	-0.928 (0.178)
	er_end	-0.997 (0.255)
	map_coef	0.903 (0.163)
	mr_end	-0.757 (0.092)
	icp_diff_begin	-0.679 (0.148)
	icp_diff_coef	-0.774 (0.175)
	icp_var_begin	0.443 (0.379)
	er_var_end	0.632 (0.193)
	mr_coef	0.574 (0.118)
	er_max_begin	-0.144 (0.099)

Abbreviations: icp, intracranial pressure; coef, regression coefficient; cpp, cerebral perfusion pressure; er, eye response; map, mean arterial pressure; mr, motor response; SD, standard deviation. For full feature abbreviation list and relative importance measures please see *Note 2 in the Electronic Supplementary Material.*

Code for the ICP-MAP-CPP algorithm

Install required Python packages

```
!conda update seaborn pandas -y
!pip install tqdm
!pip install bayesian-optimization
```

Once the packages have been installed, click Reset Session / Restart from the panel. You can also run 'Clear all Cells' from the Clear dropdown menu.

Create folder structure for images

```
!mkdir images
!mkdir images/without_gcs
!mkdir images/without_gcs/survived
!mkdir images/without_gcs/deceased
```

Load data

The data resides in the Google Cloud BigQuery data warehouse. In this section we load the data into Pandas dataframes for analysis.

```
# standard data science libraries
import pandas as pd
import numpy as np
import seaborn as sns
import matplotlib.pyplot as plt

# Google BigQuery API
import google.datalab.bigquery as bq

# libraries for HTML display and progress bars
from IPython.display import HTML
from tqdm import tqdm_notebook
```

The data is arranged into three datasets. The raw data from HUS and KYS is processed into patient_data and then combined in combined_patient_data, as explained in other notebooks.

```
%%bq datasets list
```

A quick peek into the patients dataset shows the patient ids (4-1692 for HUS patients, 12220-150050 for KYS patients and 74000-14910000 for TYKS patients), the targets dead30 and age categories.

```
%%bq query -n patients
SELECT id, agecat, dead30
FROM `combined_patient_data.patients_HUS_KYS_TYKS`
```

The monitor data with ICP and MAP are stored in a BigQuery view format with the following schema.

```
%%bq tables describe -n combined_patient_data.ICP_HUS_KYS_TYKS
%%bq query -n ICP
SELECT id, delta_icp as delta, value
FROM `combined_patient_data.ICP_HUS_KYS_TYKS`
ORDER BY id, delta
%%bq query -n MAP
SELECT id, delta_icp as delta, value
FROM `combined_patient_data.MAP_HUS_KYS_TYKS`
ORDER BY id, delta
```

CPP is then computed by the formula CPP = MAP - ICP.

```
%%bq query -n CPP
```

```
SELECT
  icp.id AS id,
  icp.delta_icp AS delta,
  map.value - icp.value AS value
FROM
  `combined_patient_data.ICP_HUS_KYS_TYKS` AS icp
INNER JOIN
  `combined_patient_data.MAP_HUS_KYS_TYKS` AS map
ON map.id = icp.id AND map.delta_icp = icp.delta_icp
ORDER BY id, delta
```

We store the query results into dataframes.

```
df_patients = patients.execute(output_options=bq.QueryOutput.dataframe()).result()
df_icp = ICP.execute(output_options=bq.QueryOutput.dataframe()).result()
df_map = MAP.execute(output_options=bq.QueryOutput.dataframe()).result()
df_cpp = CPP.execute(output_options=bq.QueryOutput.dataframe()).result()
```

Our time parameter delta is calibrated with the first ICP measurement. We therefore drop all prior measurements of MAP and CPP.

```
df_map = df_map.drop(df_map[df_map.delta < 0].index)
df_cpp = df_cpp.drop(df_cpp[df_cpp.delta < 0].index)
# Change data type of delta from float to timedelta.
df_icp.delta = df_icp.delta.map(lambda x: pd.to_timedelta(x,'s'))
df_map.delta = df_map.delta.map(lambda x: pd.to_timedelta(x,'s'))
df_cpp.delta = df_cpp.delta.map(lambda x: pd.to_timedelta(x,'s'))
```

Create features

In our case, the most crucial part in preparing the model is feature engineering. We will analyse the monitor data time series using four hour rolling windows. In these windows we compute various statistics and inspect their trends, which results in a number of derived time series. Finally, we turn these derived series into features by computing value averages over initial 24h and final 8h windows, and including a linear trend coefficient.

```
from sklearn.linear_model import LinearRegression
def create_derived_series(df_patients, df_icp, df_map, df_cpp):
  """ This function derives various new time series for each patient from the original ICP, MAP, and CPP data. """

  # Take the intersection of available patient ids.
  ids = list(set(df_patients['id']) & set(df_icp['id']) & set(df_map['id']))

  # The dictionary below holds various types of derived series as keys the values of which are dictionaries indexed by patient ids.
  # To add a new type of derived series, make and empty entry to the dictionary and provide a logic for its computation for each patient in the for loop below.
  derived_series = {'icp': {}, 'map': {}, 'cpp': {},
                    'icp_var': {}, 'map_var': {}, 'cpp_var': {},
                    'icp_ht20': {}, 'map_ht120': {}, 'icp_lt10': {},
                    'icp_diff': {}, 'map_diff': {}, 'cpp_diff': {},
                    'icp_q10': {}, 'icp_q90': {},
                    'map_q10': {}, 'map_q90': {},
                    'cpp_q10': {}, 'cpp_q90': {}}

  # Set the length of the rolling window. Feel free to experiment with values other than '4h'.
  rolling_window_length = '4h'
```

```
  # Loop over patient ids and
  for i in tqdm_notebook(ids, ncols=1000, desc="Create series"):
    # data as is
    derived_series['icp'][i] = df_icp[df_icp['id'] == i].drop('id', axis=1).set_index('delta')
    derived_series['map'][i] = df_map[df_map['id'] == i].drop('id', axis=1).set_index('delta')
    derived_series['cpp'][i] = df_cpp[df_cpp['id'] == i].drop('id', axis=1).set_index('delta')
    # variance
    derived_series['icp_var'][i] = df_icp[df_icp['id'] == i].drop('id',
axis=1).set_index('delta').rolling(rolling_window_length).var().dropna()
    derived_series['map_var'][i] = df_map[df_map['id'] == i].drop('id',
axis=1).set_index('delta').rolling(rolling_window_length).var().dropna()
    derived_series['cpp_var'][i] = df_cpp[df_cpp['id'] == i].drop('id',
axis=1).set_index('delta').rolling(rolling_window_length).var().dropna()
    # cut-off percentage
    derived_series['icp_ht20'][i] = df_icp[df_icp['id'] == i].drop('id',
axis=1).set_index('delta').rolling(rolling_window_length).apply(lambda window: 100*(window > 20).mean(),
raw=True)
    derived_series['map_ht120'][i] = df_map[df_map['id'] == i].drop('id',
axis=1).set_index('delta').rolling(rolling_window_length).apply(lambda window: 100*(window > 120).mean(),
raw=True)
    derived_series['icp_lt10'][i] = df_icp[df_icp['id'] == i].drop('id',
axis=1).set_index('delta').rolling(rolling_window_length).apply(lambda window: 100*(window < 10).mean(),
raw=True)
    # magnitude of difference
    derived_series['icp_diff'][i] = df_icp[df_icp['id'] == i].drop('id',
axis=1).set_index('delta').diff().abs().rolling(rolling_window_length).mean().dropna()
    derived_series['map_diff'][i] = df_map[df_map['id'] == i].drop('id',
axis=1).set_index('delta').diff().abs().rolling(rolling_window_length).mean().dropna()
    derived_series['cpp_diff'][i] = df_cpp[df_cpp['id'] == i].drop('id',
axis=1).set_index('delta').diff().abs().rolling(rolling_window_length).mean().dropna()
    # quantile
    derived_series['icp_q10'][i] = df_icp[df_icp['id'] == i].drop('id',
axis=1).set_index('delta').rolling(rolling_window_length).quantile(0.1).dropna()
    derived_series['icp_q90'][i] = df_icp[df_icp['id'] == i].drop('id',
axis=1).set_index('delta').rolling(rolling_window_length).quantile(0.9).dropna()
    derived_series['map_q10'][i] = df_map[df_map['id'] == i].drop('id',
axis=1).set_index('delta').rolling(rolling_window_length).quantile(0.1).dropna()
    derived_series['map_q90'][i] = df_map[df_map['id'] == i].drop('id',
axis=1).set_index('delta').rolling(rolling_window_length).quantile(0.9).dropna()
    derived_series['cpp_q10'][i] = df_cpp[df_cpp['id'] == i].drop('id',
axis=1).set_index('delta').rolling(rolling_window_length).quantile(0.1).dropna()
    derived_series['cpp_q90'][i] = df_cpp[df_cpp['id'] == i].drop('id',
axis=1).set_index('delta').rolling(rolling_window_length).quantile(0.9).dropna()

  return derived_series
```

To get an idea how the derived series look we plot one of them.

```
derived_series = create_derived_series(df_patients, df_icp, df_map, df_cpp)
name = 'icp_diff'

for i in survived:
  plt.figure(figsize=(15,5))
  plt.title('Patient {}, survived'.format(i))
  plt.plot(derived_series[name][i].index.map(lambda x: x.total_seconds() / 60 / 60), derived_series[name][i],
color='darkorange', label=name)
  plt.xlabel("Time passed (hours)")
  if name in ['icp_ht20', 'map_ht120', 'icp_lt10']:
    plt.ylabel('%')
```

```
  elif name in ['icp_var', 'map_var', 'cpp_var']:
    plt.ylabel('mmHg^2')
  else:
    plt.ylabel('mmHg')
  plt.legend(loc=1)

for i in deceased:
  plt.figure(figsize=(15,5))
  plt.title('Patient {}, deceased'.format(i))
  plt.plot(derived_series[name][i].index.map(lambda x: x.total_seconds() / 60 / 60), derived_series[name][i],
label=name)
  plt.xlabel("Time passed (hours)")
  if name in ['icp_ht20', 'map_ht120', 'icp_lt10']:
    plt.ylabel('%')
  elif name in ['icp_var', 'map_var', 'cpp_var']:
    plt.ylabel('mmHg^2')
  else:
    plt.ylabel('mmHg')
  plt.legend(loc=1)
def compute_features(series):
  """ For a given time series, compute the initial 24h mean, the final 8h mean, and the regression coefficient.
"""
  begin_mean = series[series.index < pd.to_timedelta('24h')]['value'].mean()
  end_mean = series[series.index > series.index.values[-1] - pd.to_timedelta('8h')]['value'].mean()
  coef = LinearRegression().fit(series.index.values.reshape(-1,1), series['value']).coef_[0]
  return begin_mean, end_mean, coef
def prepare_data(df_patients, df_icp, df_map, df_cpp):
  """ This function prepares the full dataframe of features from the patient and monitor data. """

  ids = list(set(df_patients['id']) & set(df_icp['id']) & set(df_map['id']))
  derived_series = create_derived_series(df_patients, df_icp, df_map, df_cpp)

  # For each type of derived series, initialize a dataframe with begin, end, and coef features.
  feature_dfs = {name: pd.DataFrame(index=ids, columns=[name + '_begin', name + '_end', name +
'_coef'], dtype=np.float32) for name in derived_series}

  series = tqdm_notebook(derived_series, ncols=1000)

  # For each type of derived series populate the corresponding feature dataframe by computing the features
for each patient.
  for name in series:
    series.set_description("%s" % name)
    for i in ids:
      try:
        feature_dfs[name].loc[i] = compute_features(derived_series[name][i])
      except:
        continue

  # Join all the feature dataframes with the patients data.
  monitordata = df_patients.set_index('id').join(feature_dfs.values(), how='inner')
  monitordata.dropna(inplace=True)
  monitordata = monitordata.sample(frac=1) # Shuffle the dataframe.
  data = monitordata.drop('dead30', axis=1)
  target = monitordata['dead30']
  return data, target
def multiprocess_prepare_data(df_patients, df_icp, df_map, df_cpp, return_dict, hour):
  """ This function prepares the full dataframe of features from the patient and monitor data. """
```

```
  ids = list(set(df_patients['id']) & set(df_icp['id']) & set(df_map['id']))
  derived_series = create_derived_series(df_patients, df_icp, df_map, df_cpp)

  # For each type of derived series, initialize a dataframe with begin, end, and coef features.
  feature_dfs = {name: pd.DataFrame(index=ids, columns=[name + '_begin', name + '_end', name +
'_coef'], dtype=np.float32) for name in derived_series}

  series = tqdm_notebook(derived_series, ncols=1000)

  # For each type of derived series populate the corresponding feature dataframe by computing the features
for each patient.
  for name in series:
    series.set_description("%s" % name)
    for i in ids:
      try:
        feature_dfs[name].loc[i] = compute_features(derived_series[name][i])
      except:
        continue

  # Join all the feature dataframes with the patients data.
  monitordata = df_patients.set_index('id').join(feature_dfs.values(), how='inner')
  monitordata.dropna(inplace=True)
  monitordata = monitordata.sample(frac=1) # Shuffle the dataframe.
  data = monitordata.drop('dead30', axis=1)
  target = monitordata['dead30']

  return_dict[hour] = data
data_full, target = prepare_data(df_patients, df_icp, df_map, df_cpp)
data_full.info()
print('Number of patients: {}'.format(len(target)))
print('Survived at least 30 days: {}'.format(len(target[target == 0.0])))
print('Deceased within 30 days: {}'.format(len(target[target == 1.0])))
print('Percentage of deceased: {}%'.format(np.round(100 * len(target[target == 1.0]) / len(target), 2)))
```

A class imbalance exists but is not critical.

Inspecting the folding methods for cross-validation.

Due to the small size of the dataset, we use 5-fold cross-validation throughout the rest of the notebook. Before proceeding further we demonstrate the outcome of a number of folding methods. See K-fold, stratified K-fold, and repeated stratified K-fold. We use stratified K-fold in order to retain the target distribution in train/test splits, and often its repeated version to average out fluctuations between splits.

```
from sklearn.model_selection import KFold, StratifiedKFold, RepeatedStratifiedKFold

#fold_method = KFold(n_splits=5, shuffle=True)
fold_method = StratifiedKFold(n_splits=5, shuffle=True)
#fold_method = RepeatedStratifiedKFold(n_splits=5, n_repeats=10)

for splitnb, (train_index, test_index) in enumerate(fold_method.split(data_full, target)):
  print('Split number {}:'.format(splitnb + 1))
  print('Number of train / test instances: {} / {}'.format(len(train_index), len(test_index)))
  print('% of deceased in train / test: {}% / {}%'.format(np.round(100 * target.iloc[train_index].mean(),2),
np.round(100 * target.iloc[test_index].mean(),2)))
  print()
```

Normalize data & select features

Normalize the data. Note that normalization is not required when using logistic regression. The

normalization procedure has been left here to enable use of other types of regressors/classifiers, such as SVM.

```
from sklearn.preprocessing import StandardScaler

scaler = StandardScaler()
data_scaled = pd.DataFrame(data=scaler.fit_transform(data_full), index=data_full.index,
columns=data_full.columns)
```

We select features using recursive feature elimination. Sets of features are evaluated by area under ROC curve in cross-validated logistic regression.

```
from sklearn.linear_model import LogisticRegression
from sklearn.feature_selection import RFECV

found = False

while not found:
  lr = LogisticRegression()

  global rfecv
  rfecv = RFECV(estimator=lr, step=1, cv=RepeatedStratifiedKFold(n_splits=5, n_repeats=10),
scoring='roc_auc')
  rfecv.fit(data_scaled, target)
  if rfecv.n_features_ >= 8 and rfecv.n_features_ <=18:
    found = True
```

Plot number of features VS. cross-validation scores

```
optimal_num_features = rfecv.n_features_
plt.figure(figsize=(16,12))
plt.title("Optimal number of features: {}".format(optimal_num_features))
plt.xlabel("Number of features selected")
plt.ylabel("Cross-validation score (ROC AUC)")
plt.plot(range(1, len(rfecv.grid_scores_) + 1), rfecv.grid_scores_)
plt.savefig('images/without_gcs/feature_count.png')
plt.show()
plt.close()
```

Select the features.

```
selected_columns = data_scaled.columns[rfecv.support_]
data = data_scaled[selected_columns]
display(HTML("Total of <b>{}</b> features considered:".format(len(data_full.columns))))
for num, column in enumerate(data_full.columns):
  print(column + '\n', file=open('images/without_gcs/tested_features.txt', 'a'))
  print(column.ljust(20), end='\t')
  if (num + 1) % 5 == 0:
    print("")
display(HTML("<b>{}</b> features selected:".format(len(data.columns))))
for num, column in enumerate(data.columns):
  print(column.ljust(20), end='\t')
  if (num + 1) % 5 == 0:
    print("")
```

We plot the correlation matrix.

```
fig, ax = plt.subplots(figsize=(25,20))
sns.set(font_scale=1.4)
sns.heatmap(data.corr(), annot=True, fmt=".2f", ax=ax, cmap="Blues")
plt.show()
sns.set(font_scale=1.0)
plt.savefig('images/without_gcs/feature_correlations.png')
plt.close()
```

Plot relative feature importances.

```
lr = LogisticRegression()
lr.fit(data, target)
feature_importance = abs(lr.coef_[0])
feature_importance = 100.0 * (feature_importance / feature_importance.max())
sorted_idx = np.argsort(feature_importance)
pos = np.arange(sorted_idx.shape[0]) + .5

featfig = plt.figure(figsize=(16,12))
featax = featfig.add_subplot(1, 1, 1)
featax.barh(pos, feature_importance[sorted_idx], align='center')
featax.set_yticks(pos)
featax.set_yticklabels(np.array(data.columns)[sorted_idx], fontsize=10)
featax.set_xlabel('Relative Feature Importance')

plt.savefig('images/without_gcs/relative_feature_importances.png')
plt.show()
plt.close()
```

Fit models and cross-validate

We proceed to fitting and evaluating a logistic regression model. We will adjust the regularization coefficient C and the class weight w by using (black-box) Bayesian optimization.

```
from sklearn.model_selection import cross_validate

def lrcv(C, w):
    scoring = ['roc_auc']
    scores = cross_validate(LogisticRegression(C=C,  class_weight={0:1,1:w}),
                            data,
                            target,
                            scoring=scoring,
                            cv=RepeatedStratifiedKFold(n_splits=5, n_repeats=10))
    val = scores['test_roc_auc'].mean()
    return val
from bayes_opt import BayesianOptimization

gp_params = {"alpha": 1e-5}

BO = BayesianOptimization(lrcv, {'C': (0.01,10), 'w': (1,1)})

BO.maximize(n_iter=20, **gp_params) # Try increasing the n_iter parameter to run the optimization longer.
print('-' * 53)

print('Final Results')
print('Classifier: %f' % BO.res['max']['max_val'])
C = BO.res['max']['max_params']['C']
w = BO.res['max']['max_params']['w']
lr = LogisticRegression(C=C, class_weight={0:1,1:w})
print(BO.res['max']['max_params'])
scoring = ['roc_auc', 'precision', 'recall', 'f1']
scores = cross_validate(lr, data, target, scoring=scoring, cv=RepeatedStratifiedKFold(n_splits=5,
n_repeats=10), return_train_score=True)
print('Averages of training scores:')
print('rocauc\tpreci\trecall\tf1')
print('{roc_auc:0.2f}\t{precision:0.2f}\t{recall:0.2f}
\t{f1:0.2f}'.format(roc_auc=scores['train_roc_auc'].mean(),
                                                    precision=scores['train_precision'].mean(),
```

```
                                                  recall=scores['train_recall'].mean(),
                                                  f1=scores['train_f1'].mean()))
print('Averages of testing scores:')
print('rocauc\tpreci\trecall\tf1')
print('{roc_auc:0.2f}\t{precision:0.2f}\t{recall:0.2f}
\t{f1:0.2f}'.format(roc_auc=scores['test_roc_auc'].mean(),
                                                  precision=scores['test_precision'].mean(),
                                                  recall=scores['test_recall'].mean(),
                                                  f1=scores['test_f1'].mean()))
```

Prediction on the full dataset

We illustrate the model performance by making predictions of the full dataset. In order to truthfully report the performance we use "cross-validated predictions". Here, for each data point the prediction is obtained from a model that was fitted without using this point.

```
from sklearn.model_selection import cross_val_predict, LeaveOneOut

pred_proba = cross_val_predict(lr, data, target, cv=LeaveOneOut(), method='predict_proba')
```

We store the outcome in a dataframe and plot both a normalized histogram and a (estimated) continuous distribution for survived and deceased separately.

```
results = pd.DataFrame()
results['true'] = target
#results['true'] = target_selected
results['pred_proba'] = pred_proba[:,1]
plt.figure(figsize=(15,5))
plt.title("Prediction probabilites survived & deceased (normalized histogram)")
plt.ylabel("Density")
sns.distplot(results[results['true'] == 1]['pred_proba'], bins=10, kde=False, label='deceased',
norm_hist=True)
sns.distplot(results[results['true'] == 0]['pred_proba'], bins=10, kde=False, label='survived',
norm_hist=True)
plt.xlabel("Predicted probability")
plt.legend()
plt.figure(figsize=(15,5))
plt.title("Prediction probabilites survived & deceased (kde)")
plt.ylabel("Density")
sns.distplot(results[results['true'] == 1]['pred_proba'], bins=10, hist=False, label='deceased')
sns.distplot(results[results['true'] == 0]['pred_proba'], bins=10, hist=False, label='survived')
plt.xlabel("Predicted probability")
plt.legend()
```

In order to make strict classifications and compute the accuracy, we set a threshold.

```
threshold = 0.5
results['pred'] = 1 * (results['pred_proba'] > threshold)
```

The misclassifications are examined below.

```
results[results['true'] != results['pred']].to_csv('images/without_gcs/misclassifications.csv')
with pd.option_context('display.max_rows', None, 'display.max_columns', None):
    print(results[results['true'] != results['pred']])
from sklearn.metrics import confusion_matrix, accuracy_score

print('Accuracy: {}%'.format(np.round(100 * accuracy_score(results['true'], results['pred']), 2)))
print()
print('Confusion matrix:')
cm = confusion_matrix(results['true'], results['pred'])
print(cm)
print()
```

```
print("False positives: {}".format(cm[0][1]))
print("False negatives: {}".format(cm[1][0]))
```

Calculate features for each time window (multiprocess)

```
from sklearn.preprocessing import StandardScaler

# Calculate features for the full dataset
data_full, target = prepare_data(df_patients, df_icp, df_map, df_cpp)

# Scale the features
scaler = StandardScaler()
data_scaled = pd.DataFrame(data=scaler.fit_transform(data_full), index=data_full.index,
columns=data_full.columns)

# Sort indices
data = data_scaled.sort_index()
target = target.sort_index()

# Create dictionaries for the features
data_full_pred = dict()  # All the features computed from truncated time series
data_pred = dict()  # All the scaled features

import multiprocessing as mp

hours = range(24, 128, 8)
manager = mp.Manager()
data_full_pred = manager.dict()
n_cores = 4

# created pool running maximum 4 cores
pool = mp.Pool(n_cores)

# Execute the feature calculation in parallel
for hour in hours:
    pool.apply_async(multiprocess_prepare_data, args=(df_patients,
                                                     df_icp[df_icp.delta < str(hour) + "h"],
                                                     df_map[df_map.delta < str(hour) + "h"],
                                                     df_cpp[df_cpp.delta < str(hour) + "h"],
                                                     data_full_pred,
                                                     hour))

# Tell the pool that there are no more tasks to come and join
pool.close()
pool.join()

for hour in hours:
  data_pred[hour] = pd.DataFrame(data=scaler.transform(data_full_pred[hour]),
index=data_full_pred[hour].index, columns=data_full_pred[hour].columns)
  data_pred[hour] = data_pred[hour].sort_index()
```

Calculate cross-validated AUC-ROC

```
from sklearn.model_selection import KFold, StratifiedKFold, RepeatedStratifiedKFold
from sklearn.metrics import roc_curve, auc, roc_auc_score
from scipy import interp

fold_method = RepeatedStratifiedKFold(n_splits=5, n_repeats=20)
lr_model = LogisticRegression(C=C, class_weight={0:1,1:w})
```

```
aucs_train = dict()
aucs_test = dict()
for hours in range(24, 128, 8):
  aucs_train[hours] = list()
  aucs_test[hours] = list()

selected_columns = data.columns[rfecv.support_]
data = data[selected_columns]

for train_indices, test_indices in fold_method.split(data, target):
  # train_indices and test_indices are positional indices, transforming them to patient_ids:
  train_patients, test_patients = data.index[train_indices], data.index[test_indices]

  # Train the fold specific model with full time series
  classifier = lr_model.fit(data.loc[train_patients], target.loc[train_patients])

  # Calculate metrics for each time window
  for hours in range(24, 128, 8):
    preds_train = classifier.predict_proba(data_pred[hours][selected_columns].loc[train_patients])[:,1]
    preds_test = classifier.predict_proba(data_pred[hours][selected_columns].loc[test_patients])[:,1]

    score_train = roc_auc_score(target.loc[train_patients], preds_train)
    score_test = roc_auc_score(target.loc[test_patients], preds_test)

    aucs_train[hours].append(score_train)
    aucs_test[hours].append(score_test)
```

Calculate hourly AUC means, AUC standard deviations and plot hourly AUCs with their error estimates

```
def auc_stats(aucs):
  aucs_mean = []
  aucs_std = []
  aucs_lower = []
  aucs_upper = []

  for hours in range(24, 128, 8):
    mean = np.mean(aucs[hours])
    aucs_mean.append(mean)

    std = np.std(aucs[hours])
    aucs_std.append(std)

    lower = mean - std
    aucs_lower.append(lower)

    upper = mean + std
    aucs_upper.append(upper)

  return aucs_mean, aucs_lower, aucs_upper

aucs_train_mean, aucs_train_lower, aucs_train_upper = auc_stats(aucs_train)
aucs_test_mean, aucs_test_lower, aucs_test_upper = auc_stats(aucs_test)

time = range(24, 128, 8)

plt.figure(figsize=(16,12))
plt.plot(time, aucs_train_mean, color='black', linestyle='--', label=r'AUROC $\pm$ 1 std. dev. (train)')
```

```
plt.plot(time, aucs_test_mean, color='red', label=r'AUROC $\pm$ 1 std. dev. (validation)')
plt.fill_between(time, aucs_train_lower, aucs_train_upper, color='black', alpha=.2)
plt.fill_between(time, aucs_test_lower, aucs_test_upper, color='red', alpha=.1)
plt.xlabel('Time (h)')
plt.ylabel('Area under ROC')
plt.legend(loc='upper right')
plt.ylim((0.6,1.0))

plt.savefig('images/without_gcs/auc_roc.png')
plt.show()
```

View the predictions dynamically

One of the central requirements for our model was to have it predict dynamically, i.e. to make it sensitive to changes in the ICP-MAP measurements for each patient. The features facilitating this are naturally the final 8h means of each derived series as well as their linear trend coefficients. Below we illustrate this by rolling out the monitor data in 8 hour windows and predicting as we go.

Notice that the model is fit on the full dataset of untruncated time series. While the rolled out monitor data is in principle unseen to the model, some features such as the initial 24h means do not change. One should therefore view this primarily as an illustration and not a test.

Computing the features for each 8h step takes a few minutes.

```
lr = LogisticRegression(C=C, class_weight={0:1,1:w})
lr.fit(data[selected_columns], target)

predict_proba = dict()
all_proba = pd.DataFrame() # a dataframe for the predictions
all_proba['true'] = target

for hours in tqdm_notebook(range(24, 128, 8), ncols=1000, desc="Hours"):
  predict_proba[hours] = lr.predict_proba(data_pred[hours][selected_columns])[:,1] # the slicing chooses the probability of death for each patient
  pred = pd.DataFrame(predict_proba[hours], index=data_pred[hours].index)
  all_proba[hours] = pred
proba_dead = all_proba[all_proba.true == 1].drop(columns='true').transpose().sort_index()
proba_alive = all_proba[all_proba.true == 0].drop(columns='true').transpose().sort_index()
df_icp['hours'] = df_icp['delta'].map(lambda x: x.total_seconds() / 60 / 60)
df_map['hours'] = df_map['delta'].map(lambda x: x.total_seconds() / 60 / 60)
df_cpp['hours'] = df_cpp['delta'].map(lambda x: x.total_seconds() / 60 / 60)
```

It is interesting to view how the predictions evolve for patients who deceased. We plot the predictions on top of their ICP-MAP-CPP data.

```
for i in proba_dead.columns:
  fig, ax1 = plt.subplots()

  fig.set_size_inches(25, 5)
  plt.title('Patient {} deceased'.format(i))
  plt.xlabel("Time passed (hours)")
  p0, = ax1.plot((proba_dead.index), proba_dead[i], label="estimate", color='red')
  ax1.set_ylim([0,1.01])
  ax1.set_ylabel('Estimate', fontsize=12)
  ax1.tick_params('y', colors='red')
  ax1.grid(visible=False)

  ax2 = ax1.twinx()
  p1, = ax2.plot(df_icp[df_icp.id == i].hours, df_icp[df_icp.id == i].value, label='icp')
  p2, = ax2.plot(df_map[df_map.id == i].hours, df_map[df_map.id == i].value, label='map')
```

```
    p3, = ax2.plot(df_cpp[df_cpp.id == i].hours, df_cpp[df_cpp.id == i].value, label='cpp')
    ax2.set_ylim([0, 150])
    ax2.set_ylabel('mmHg', fontsize=12)

    lines = [p0, p1, p2, p3]

    ax2.legend(lines, [l.get_label() for l in lines], loc=2)

    fig.tight_layout()
    fig.savefig('images/without_gcs/deceased/' + str(i) + '_timeseries.png')
    plt.close()
for i in proba_alive.columns:
    fig, ax1 = plt.subplots()

    fig.set_size_inches(25, 5)
    plt.title('Patient {} survived'.format(i))
    plt.xlabel("Time passed (hours)")
    p0, = ax1.plot((proba_alive.index), proba_alive[i], label="estimate", color='red')
    ax1.set_ylim([0,1.01])
    ax1.set_ylabel('Estimate', fontsize=12)
    ax1.tick_params('y', colors='red')
    ax1.grid(visible=False)

    ax2 = ax1.twinx()
    p1, = ax2.plot(df_icp[df_icp.id == i].hours, df_icp[df_icp.id == i].value, label='icp')
    p2, = ax2.plot(df_map[df_map.id == i].hours, df_map[df_map.id == i].value, label='map')
    p3, = ax2.plot(df_cpp[df_cpp.id == i].hours, df_cpp[df_cpp.id == i].value, label='cpp')
    ax2.set_ylim([0, 150])
    ax2.set_ylabel('mmHg', fontsize=12)

    lines = [p0, p1, p2, p3]

    ax2.legend(lines, [l.get_label() for l in lines], loc=2)

    fig.tight_layout()
    fig.savefig('images/without_gcs/survived/' + str(i) + '_timeseries.png')
    plt.close()
```

Let's take a closer look at our model.

```
from matplotlib.ticker import FixedFormatter

i = np.random.choice(proba_dead.columns)

importance_dict = {}
for hour in proba_dead.index:
    importance_dict[hour] = data_pred[hour][selected_columns].loc[i] * lr.coef_[0]

plt.figure(figsize=(25,5))
sns.heatmap(np.array([importance_dict[hour] for hour in proba_dead.index]).T,
            linewidth=0.1,
            cmap=sns.color_palette("coolwarm", 6),
            #cmap=sns.cubehelix_palette(n_colors=6, as_cmap=False),
            #cbar_kws={'label': 'indication', 'ticks': [-0.82,-0.5,-0.16,0.16,0.5,0.82], 'format': 
FixedFormatter(['extremely good','good','slightly good','slightly bad','bad','extremely bad'])},
            #vmin=-1, vmax=1,
            #cbar_kws={'label': 'indication', 'ticks': [-1.65,-1.0,-0.35,0.35,1.0,1.65], 'format': 
FixedFormatter(['extremely good','good','slightly good','slightly bad','bad','extremely bad'])},
```

```
        #vmin=-2, vmax=2,
        #cbar_kws={'label': 'indication', 'ticks': [-1.65,-1.0,-0.35,0.35,1.0,1.65], 'format':
FixedFormatter(['extremely good','good','slightly good','slightly bad','bad','extremely bad'])},
        #vmin=-2, vmax=2,
        cbar_kws={'label': 'indication', 'ticks': [-2.5,-1.5,-0.5,0.5,1.5,2.5], 'format':
FixedFormatter(['extremely good','good','slightly good','slightly bad','bad','extremely bad'])},
        vmin=-3, vmax=3,
        #cbar_kws={'label': 'indication', 'ticks': [-3.3,-2.0,-0.7,0.7,2.0,3.3], 'format':
FixedFormatter(['extremely good','good','slightly good','slightly bad','bad','extremely bad'])},
        #vmin=-4, vmax=4,
        #cbar_kws={'label': 'indication', 'ticks': [-4.1,-2.5,-0.8,0.8,2.5,4.1], 'format':
FixedFormatter(['extremely good','good','slightly good','slightly bad','bad','extremely bad'])},
        #vmin=-5, vmax=5,
        xticklabels=[str(hour) + ': ' + str(int(np.rint(100 * proba_dead[i][hour]))) + '%' for hour in
proba_dead.index],
        yticklabels=importance_dict[24].keys())
plt.title('Patient {} deceased'.format(i))
plt.xlabel('Time passed (hours): Estimated probability of death')
from matplotlib.ticker import FixedFormatter

for i in proba_dead.columns:
  importance_dict = {}
  for hour in proba_dead.index:
    importance_dict[hour] = data_pred[hour][selected_columns].loc[i] * lr.coef_[0]

  plt.figure(figsize=(25,5))
  sns.heatmap(np.array([importance_dict[hour] for hour in proba_dead.index]).T,
          linewidth=0.1,
          cmap=sns.color_palette("coolwarm", 6),
          #cmap=sns.cubehelix_palette(n_colors=6, as_cmap=False),
          #cbar_kws={'label': 'indication', 'ticks': [-0.82,-0.5,-0.16,0.16,0.5,0.82], 'format':
FixedFormatter(['extremely good','good','slightly good','slightly bad','bad','extremely bad'])},
          #vmin=-1, vmax=1,
          #cbar_kws={'label': 'indication', 'ticks': [-1.65,-1.0,-0.35,0.35,1.0,1.65], 'format':
FixedFormatter(['extremely good','good','slightly good','slightly bad','bad','extremely bad'])},
          #vmin=-2, vmax=2,
          #cbar_kws={'label': 'indication', 'ticks': [-1.65,-1.0,-0.35,0.35,1.0,1.65], 'format':
FixedFormatter(['extremely good','good','slightly good','slightly bad','bad','extremely bad'])},
          #vmin=-2, vmax=2,
          cbar_kws={'label': 'indication', 'ticks': [-2.5,-1.5,-0.5,0.5,1.5,2.5], 'format':
FixedFormatter(['extremely good','good','slightly good','slightly bad','bad','extremely bad'])},
          vmin=-3, vmax=3,
          #cbar_kws={'label': 'indication', 'ticks': [-3.3,-2.0,-0.7,0.7,2.0,3.3], 'format':
FixedFormatter(['extremely good','good','slightly good','slightly bad','bad','extremely bad'])},
          #vmin=-4, vmax=4,
          #cbar_kws={'label': 'indication', 'ticks': [-4.1,-2.5,-0.8,0.8,2.5,4.1], 'format':
FixedFormatter(['extremely good','good','slightly good','slightly bad','bad','extremely bad'])},
          #vmin=-5, vmax=5,
          xticklabels=[str(hour) + ': ' + str(int(np.rint(100 * proba_dead[i][hour]))) + '%' for hour in
proba_dead.index],
          yticklabels=importance_dict[24].keys())
  plt.title('Patient {} deceased'.format(i))
  plt.xlabel('Time passed (hours): Estimated probability of death')
  plt.savefig('images/without_gcs/deceased/' + str(i) + '_featureimportance.png')
  plt.close()
from matplotlib.ticker import FixedFormatter

for i in proba_alive.columns:
```

```
importance_dict = {}
for hour in proba_alive.index:
  importance_dict[hour] = data_pred[hour][selected_columns].loc[i] * lr.coef_[0]

plt.figure(figsize=(25,5))
sns.heatmap(np.array([importance_dict[hour] for hour in proba_alive.index]).T,
            linewidth=0.1,
            cmap=sns.color_palette("coolwarm", 6),
            #cmap=sns.cubehelix_palette(n_colors=6, as_cmap=False),
            #cbar_kws={'label': 'indication', 'ticks': [-0.82,-0.5,-0.16,0.16,0.5,0.82], 'format':
FixedFormatter(['extremely good','good','slightly good','slightly bad','bad','extremely bad'])},
            #vmin=-1, vmax=1,
            #cbar_kws={'label': 'indication', 'ticks': [-1.65,-1.0,-0.35,0.35,1.0,1.65], 'format':
FixedFormatter(['extremely good','good','slightly good','slightly bad','bad','extremely bad'])},
            #vmin=-2, vmax=2,
            #cbar_kws={'label': 'indication', 'ticks': [-1.65,-1.0,-0.35,0.35,1.0,1.65], 'format':
FixedFormatter(['extremely good','good','slightly good','slightly bad','bad','extremely bad'])},
            #vmin=-2, vmax=2,
            cbar_kws={'label': 'indication', 'ticks': [-2.5,-1.5,-0.5,0.5,1.5,2.5], 'format':
FixedFormatter(['extremely good','good','slightly good','slightly bad','bad','extremely bad'])},
            vmin=-3, vmax=3,
            #cbar_kws={'label': 'indication', 'ticks': [-3.3,-2.0,-0.7,0.7,2.0,3.3], 'format':
FixedFormatter(['extremely good','good','slightly good','slightly bad','bad','extremely bad'])},
            #vmin=-4, vmax=4,
            #cbar_kws={'label': 'indication', 'ticks': [-4.1,-2.5,-0.8,0.8,2.5,4.1], 'format':
FixedFormatter(['extremely good','good','slightly good','slightly bad','bad','extremely bad'])},
            #vmin=-5, vmax=5,
            xticklabels=[str(hour) + ': ' + str(int(np.rint(100 * proba_alive[i][hour]))) + '%' for hour in
proba_alive.index],
            yticklabels=importance_dict[24].keys())
plt.title('Patient {} survived'.format(i))
plt.xlabel('Time passed (hours): Estimated probability of death')
plt.savefig('images/without_gcs/survived/' + str(i) + '_featureimportance.png')
plt.close()
```

Model feature importances

```
print('Feature coefficients:')
dict(zip(data.columns, np.round(lr.coef_[0],2)))
plt.figure()
plt.title('Predictive role of (high numerical values of) features.')
#ticks = [-0.9,-0.3,0.3,0.9]
#vmin = -1.2
#vmax = 1.2
ticks = [-1.2,-0.4,0.4,1.2]
vmin = -1.6
vmax = 1.6
sns.heatmap(np.array([lr.coef_[0]]).T,
           linewidth=0.1,
           cmap=sns.color_palette("coolwarm", 4),
           cbar_kws={'label': 'predictor', 'ticks': ticks, 'format': FixedFormatter(['good','slightly good','slightly
bad','bad',])},
           vmin=vmin, vmax=vmax,
           xticklabels=[],
           yticklabels=importance_dict[24].keys(),
           square=True)
plt.savefig('images/without_gcs/overall_feature_importances.png')
```

Evolution of prediction distribution over time

```
all_proba_plot = all_proba.copy()

while all_proba_plot.iloc[0]['true'] == 0:
  all_proba_plot = all_proba_plot.sample(frac=1)
fig = plt.figure(figsize=(25,10))
fig.suptitle('Predicted scores for survived / deceased by hour.')

plot_data_1 = pd.melt(all_proba_plot, id_vars=['true'], value_vars=[24, 32, 40, 48, 56, 64], var_name='hour',
value_name='score')
plot_data_1['true'] = plot_data_1['true'].map({0.0: 'survived', 1.0: 'deceased'})

plot_data_2 = pd.melt(all_proba_plot, id_vars=['true'], value_vars=[72, 80, 88, 96, 104, 112, 120],
var_name='hour', value_name='score')
plot_data_2['true'] = plot_data_2['true'].map({0.0: 'survived', 1.0: 'deceased'})

plt.subplot(2,1,1)
sns.violinplot(x="hour", y="score", hue="true", cut=0, scale='count', bw=0.2, data=plot_data_1, split=True)

plt.subplot(2,1,2)
sns.violinplot(x="hour", y="score", hue="true", cut=0, scale='count', bw=0.2, data=plot_data_2, split=True)
plt.savefig('images/without_gcs/violinplot.png')
```

Copy all images to Google Cloud Bucket

```
!gsutil -m cp -r images/without_gcs/ gs://hus-aip-tbi/images/
```

Code for the ICP-MAP-CPP-GCS algorithm

Install required Python packages

```
!conda update seaborn pandas -y
!pip install tqdm
!pip install bayesian-optimization
```

Once the packages have been installed, click Reset Session / Restart from the panel. You can also run 'Clear all Cells' from the Clear dropdown menu.

Create folder structure for images

```
!mkdir images
!mkdir images/with_gcs
!mkdir images/with_gcs/survived
!mkdir images/with_gcs/deceased
```

Load data

The data resides in the Google Cloud BigQuery data warehouse. In this section we load the data into Pandas dataframes for analysis.

```
# standard data science libraries
import pandas as pd
import numpy as np
import seaborn as sns
import matplotlib.pyplot as plt
# Google BigQuery API
import google.datalab.bigquery as bq
# libraries for HTML display and progress bars
from IPython.display import HTML
from tqdm import tqdm_notebook
```

The data is arranged into three datasets. The raw data from HUS and KYS is processed into patient_data and then combined in combined_patient_data, as explained in other notebooks.

```
%%bq datasets list
```

A quick peek into the patients dataset shows the patient ids (4-1692 for HUS patients, 12220-150050 for KYS patients and 74000-14910000 for TYKS patients), the targets dead30 and age categories.

```
%%bq query -n patients
SELECT id, agecat, dead30
FROM `combined_patient_data.patients_HUS_KYS_TYKS`
```

The monitor data with ICP and MAP are stored in a BigQuery view format with the following schema.

```
%%bq tables describe -n combined_patient_data.ICP_HUS_KYS_TYKS
%%bq query -n ICP
SELECT id, delta_icp as delta, value
FROM `combined_patient_data.ICP_HUS_KYS_TYKS`
ORDER BY id, delta
%%bq query -n MAP
SELECT id, delta_icp as delta, value
FROM `combined_patient_data.MAP_HUS_KYS_TYKS`
ORDER BY id, delta
```

CPP is then computed by the formula CPP = MAP - ICP.

```
%%bq query -n CPP
SELECT
  icp.id AS id,
  icp.delta_icp AS delta,
  map.value - icp.value AS value
FROM
  `combined_patient_data.ICP_HUS_KYS_TYKS` AS icp
INNER JOIN
  `combined_patient_data.MAP_HUS_KYS_TYKS` AS map
ON map.id = icp.id AND map.delta_icp = icp.delta_icp
```

```
ORDER BY id, delta
%%bq query -n GCS
SELECT id, delta_icp as delta, motor_response, eye_response
FROM `combined_patient_data.GCS_HUS_KYS_TYKS`
ORDER BY id, delta
```

We store the query results into dataframes.

```
df_patients = patients.execute(output_options=bq.QueryOutput.dataframe()).result()
df_icp = ICP.execute(output_options=bq.QueryOutput.dataframe()).result()
df_map = MAP.execute(output_options=bq.QueryOutput.dataframe()).result()
df_cpp = CPP.execute(output_options=bq.QueryOutput.dataframe()).result()
df_gcs = GCS.execute(output_options=bq.QueryOutput.dataframe()).result()
```

Our time parameter delta is calibrated with the first ICP measurement. We therefore drop all prior measurements of MAP and CPP.

```
df_map = df_map.drop(df_map[df_map.delta < 0].index)
df_cpp = df_cpp.drop(df_cpp[df_cpp.delta < 0].index)
df_gcs = df_gcs.drop(df_gcs[df_gcs.delta < 0].index)
# Change data type of delta from float to timedelta.
df_icp.delta = df_icp.delta.map(lambda x: pd.to_timedelta(x,'s'))
df_map.delta = df_map.delta.map(lambda x: pd.to_timedelta(x,'s'))
df_cpp.delta = df_cpp.delta.map(lambda x: pd.to_timedelta(x,'s'))
df_gcs.delta = df_gcs.delta.map(lambda x: pd.to_timedelta(x,'s'))
df_mr = df_gcs.drop(columns='eye_response')
df_mr.columns = ['id', 'delta', 'value']
df_er = df_gcs.drop(columns='motor_response')
df_er.columns = ['id', 'delta', 'value']
```

Create features

In our case, the most crucial part in preparing the model is feature engineering. We will analyse the monitor data time series using four hour rolling windows. In these windows we compute various statistics and inspect their trends, which results in a number of derived time series. Finally, we turn these derived series into features by computing value averages over initial 24h and final 8h windows, and including a linear trend coefficient.

```
from sklearn.linear_model import LinearRegression
def create_derived_series(df_patients, df_icp, df_map, df_cpp, df_mr, df_er):
  """ This function derives various new time series for each patient from the original ICP, MAP, CPP, and GCS data.
"""

  # Take the intersection of available patient ids.
  ids = list(set(df_patients['id']) & set(df_icp['id']) & set(df_map['id']) & set(df_mr['id']) & set(df_er['id']))

  # The dictionary below holds various types of derived series as keys the values of which are dictionaries indexed by patient ids.
  # To add a new type of derived series, make and empty entry to the dictionary and provide a logic for its computation for each patient in the for loop below.
  derived_series = {'icp': {}, 'map': {}, 'cpp': {}, 'mr': {}, 'er': {},
                    'icp_var': {}, 'map_var': {}, 'cpp_var': {}, 'mr_var': {}, 'er_var': {},
                    'icp_ht20': {}, 'map_ht120': {}, 'icp_lt10': {},
                    'icp_diff': {}, 'map_diff': {}, 'cpp_diff': {},
                    'icp_q10': {}, 'icp_q90': {},
                    'map_q10': {}, 'map_q90': {},
                    'cpp_q10': {}, 'cpp_q90': {},
                    'er_min': {},
                    'er_max': {},
                    'er_avg': {}}

  # Set the length of the rolling window. Feel free to experiment with values other than '4h'.
  rolling_window_length = '4h'
  gcs_rolling_window_length = '24h'


  # Loop over patient ids and
  for i in tqdm_notebook(ids, ncols=1000, desc="Create series"):
    # data as is
```

```
    derived_series['icp'][i] = df_icp[df_icp['id'] == i].drop('id', axis=1).set_index('delta')
    derived_series['map'][i] = df_map[df_map['id'] == i].drop('id', axis=1).set_index('delta')
    derived_series['cpp'][i] = df_cpp[df_cpp['id'] == i].drop('id', axis=1).set_index('delta')
    derived_series['mr'][i] = df_mr[df_mr['id'] == i].drop('id', axis=1).set_index('delta')
    derived_series['er'][i] = df_er[df_er['id'] == i].drop('id', axis=1).set_index('delta')
    # variance
    derived_series['icp_var'][i] = df_icp[df_icp['id'] == i].drop('id',
axis=1).set_index('delta').rolling(rolling_window_length).var().dropna()
    derived_series['map_var'][i] = df_map[df_map['id'] == i].drop('id',
axis=1).set_index('delta').rolling(rolling_window_length).var().dropna()
    derived_series['cpp_var'][i] = df_cpp[df_cpp['id'] == i].drop('id',
axis=1).set_index('delta').rolling(rolling_window_length).var().dropna()
    derived_series['mr_var'][i] = df_mr[df_mr['id'] == i].drop('id',
axis=1).set_index('delta').rolling(gcs_rolling_window_length).var().dropna()
    derived_series['er_var'][i] = df_er[df_er['id'] == i].drop('id',
axis=1).set_index('delta').rolling(gcs_rolling_window_length).var().dropna()
    # cut-off percentage
    derived_series['icp_ht20'][i] = df_icp[df_icp['id'] == i].drop('id',
axis=1).set_index('delta').rolling(rolling_window_length).apply(lambda window: 100*(window > 20).mean(),
raw=True)
    derived_series['map_ht120'][i] = df_map[df_map['id'] == i].drop('id',
axis=1).set_index('delta').rolling(rolling_window_length).apply(lambda window: 100*(window > 120).mean(),
raw=True)
    derived_series['icp_lt10'][i] = df_icp[df_icp['id'] == i].drop('id',
axis=1).set_index('delta').rolling(rolling_window_length).apply(lambda window: 100*(window < 10).mean(),
raw=True)
    # magnitude of difference
    derived_series['icp_diff'][i] = df_icp[df_icp['id'] == i].drop('id',
axis=1).set_index('delta').diff().abs().rolling(rolling_window_length).mean().dropna()
    derived_series['map_diff'][i] = df_map[df_map['id'] == i].drop('id',
axis=1).set_index('delta').diff().abs().rolling(rolling_window_length).mean().dropna()
    derived_series['cpp_diff'][i] = df_cpp[df_cpp['id'] == i].drop('id',
axis=1).set_index('delta').diff().abs().rolling(rolling_window_length).mean().dropna()
    # quantile
    derived_series['icp_q10'][i] = df_icp[df_icp['id'] == i].drop('id',
axis=1).set_index('delta').rolling(rolling_window_length).quantile(0.1).dropna()
    derived_series['icp_q90'][i] = df_icp[df_icp['id'] == i].drop('id',
axis=1).set_index('delta').rolling(rolling_window_length).quantile(0.9).dropna()
    derived_series['map_q10'][i] = df_map[df_map['id'] == i].drop('id',
axis=1).set_index('delta').rolling(rolling_window_length).quantile(0.1).dropna()
    derived_series['map_q90'][i] = df_map[df_map['id'] == i].drop('id',
axis=1).set_index('delta').rolling(rolling_window_length).quantile(0.9).dropna()
    derived_series['cpp_q10'][i] = df_cpp[df_cpp['id'] == i].drop('id',
axis=1).set_index('delta').rolling(rolling_window_length).quantile(0.1).dropna()
    derived_series['cpp_q90'][i] = df_cpp[df_cpp['id'] == i].drop('id',
axis=1).set_index('delta').rolling(rolling_window_length).quantile(0.9).dropna()
    # minumum
    derived_series['er_min'][i] = df_er[df_er['id'] == i].drop('id',
axis=1).set_index('delta').rolling(gcs_rolling_window_length).min().dropna()
    # maximum
    derived_series['er_max'][i] = df_er[df_er['id'] == i].drop('id',
axis=1).set_index('delta').rolling(gcs_rolling_window_length).max().dropna()
    # average
    derived_series['er_avg'][i] = df_er[df_er['id'] == i].drop('id',
axis=1).set_index('delta').rolling(gcs_rolling_window_length).mean().dropna()

  return derived_series
def compute_features(series):
  """ For a given time series, compute the initial 24h mean, the final 8h mean, and the regression coefficient. """
  begin_mean = series[series.index < pd.to_timedelta('24h')]['value'].mean()
  end_mean = series[series.index > series.index.values[-1] - pd.to_timedelta('8h')]['value'].mean()
  coef = LinearRegression().fit(series.index.values.reshape(-1,1), series['value']).coef_[0]
  return begin_mean, end_mean, coef
def prepare_data(df_patients, df_icp, df_map, df_cpp, df_mr, df_er):
  """ This function prepares the full dataframe of features from the patient and monitor data. """
```

```
  ids = list(set(df_patients['id']) & set(df_icp['id']) & set(df_map['id']) & set(df_mr['id']) & set(df_er['id']))
  derived_series = create_derived_series(df_patients, df_icp, df_map, df_cpp, df_mr, df_er)

  # For each type of derived series, initialize a dataframe with begin, end, and coef features.
  feature_dfs = {name: pd.DataFrame(index=ids, columns=[name + '_begin', name + '_end', name + '_coef'],
dtype=np.float32) for name in derived_series}

  series = tqdm_notebook(derived_series, ncols=1000)

  # For each type of derived series populate the corresponding feature dataframe by computing the features for each
patient.
  for name in series:
    series.set_description("%s" % name)
    for i in ids:
      try:
        feature_dfs[name].loc[i] = compute_features(derived_series[name][i])
      except:
        continue


  # Join all the feature dataframes with the patients data.
  monitordata = df_patients.set_index('id').join(feature_dfs.values(), how='inner')
  monitordata.dropna(inplace=True)
  monitordata = monitordata.sample(frac=1) # Shuffle the dataframe.
  data = monitordata.drop('dead30', axis=1)
  target = monitordata['dead30']
  return data, target
def multiprocess_prepare_data(df_patients, df_icp, df_map, df_cpp, df_mr, df_er, return_dict, hour):
  """ This function prepares the full dataframe of features from the patient and monitor data. """

  ids = list(set(df_patients['id']) & set(df_icp['id']) & set(df_map['id']) & set(df_mr['id']) & set(df_er['id']))
  derived_series = create_derived_series(df_patients, df_icp, df_map, df_cpp, df_mr, df_er)

  # For each type of derived series, initialize a dataframe with begin, end, and coef features.
  feature_dfs = {name: pd.DataFrame(index=ids, columns=[name + '_begin', name + '_end', name + '_coef'],
dtype=np.float32) for name in derived_series}

  series = tqdm_notebook(derived_series, ncols=1000)

  # For each type of derived series populate the corresponding feature dataframe by computing the features for each
patient.
  for name in series:
    series.set_description("%s" % name)
    for i in ids:
      try:
        feature_dfs[name].loc[i] = compute_features(derived_series[name][i])
      except:
        continue

  # Join all the feature dataframes with the patients data.
  monitordata = df_patients.set_index('id').join(feature_dfs.values(), how='inner')
  monitordata.dropna(inplace=True)
  monitordata = monitordata.sample(frac=1) # Shuffle the dataframe.
  data = monitordata.drop('dead30', axis=1)
  target = monitordata['dead30']

  return_dict[hour] = data
data_full, target = prepare_data(df_patients, df_icp, df_map, df_cpp, df_mr, df_er)
data_full.info()
print('Number of patients: {}'.format(len(target)))
print('Survived at least 30 days: {}'.format(len(target[target == 0.0])))
print('Deceased within 30 days: {}'.format(len(target[target == 1.0])))
print('Percentage of deceased: {}%'.format(np.round(100 * len(target[target == 1.0]) / len(target), 2)))
```

A class imbalance exists but is not critical.

Inspecting the folding methods for cross-validation.

Due to the small size of the dataset, we use 5-fold cross-validation throughout the rest of the notebook. Before proceeding further we demonstrate the outcome of a number of folding methods. See K-fold, stratified K-fold, and repeated stratified K-fold. We use stratified K-fold in order to retain the target distribution in train/test splits, and often its repeated version to average out fluctuations between splits.

```
from sklearn.model_selection import KFold, StratifiedKFold, RepeatedStratifiedKFold
#fold_method = KFold(n_splits=5, shuffle=True)
fold_method = StratifiedKFold(n_splits=5, shuffle=True)
#fold_method = RepeatedStratifiedKFold(n_splits=5, n_repeats=10)
for splitnb, (train_index, test_index) in enumerate(fold_method.split(data_full, target)):
  print('Split number {}:'.format(splitnb + 1))
  print('Number of train / test instances: {} / {}'.format(len(train_index), len(test_index)))
  print('% of deceased in train / test: {}% / {}%'.format(np.round(100 * target.iloc[train_index].mean(),2), np.round(100
* target.iloc[test_index].mean(),2)))
  print()
```

Normalize data & select features

Normalize the data. Note that normalization is not required when using logistic regression. The normalization procedure has been left here to enable use of other types of regressors/classifiers, such as SVM.

```
from sklearn.preprocessing import StandardScaler
scaler = StandardScaler()
data_scaled = pd.DataFrame(data=scaler.fit_transform(data_full), index=data_full.index, columns=data_full.columns)
```

We select features using recursive feature elimination. Sets of features are evaluated by area under ROC curve in cross-validated logistic regression.

```
from sklearn.linear_model import LogisticRegression
from sklearn.feature_selection import RFECV
found = False
while not found:
  lr = LogisticRegression()

  global rfecv
  rfecv = RFECV(estimator=lr, step=1, cv=RepeatedStratifiedKFold(n_splits=5, n_repeats=10), scoring='roc_auc')
  rfecv.fit(data_scaled, target)
  if rfecv.n_features_ >= 14 and rfecv.n_features_ <=14:
    found = True
```

Plot number of features VS. cross-validation scores

```
optimal_num_features = rfecv.n_features_
plt.figure(figsize=(16,12))
plt.title("Optimal number of features: {}".format(optimal_num_features))
plt.xlabel("Number of features selected")
plt.ylabel("Cross-validation score (ROC AUC)")
plt.plot(range(1, len(rfecv.grid_scores_) + 1), rfecv.grid_scores_)
plt.savefig('images/with_gcs/feature_count.png')
plt.show()
plt.close()
```

Select the features.

```
selected_columns = data_scaled.columns[rfecv.support_]
data = data_scaled[selected_columns]
display(HTML("Total of <b>{}</b> features considered:".format(len(data_full.columns))))
for num, column in enumerate(data_full.columns):
  print(column + '\n', file=open('images/with_gcs/tested_features.txt', 'a'))
  print(column.ljust(20), end='\t')
  if (num + 1) % 5 == 0:
    print("")
display(HTML("<b>{}</b> features selected:".format(len(data.columns))))
for num, column in enumerate(data.columns):
  print(column.ljust(20), end='\t')
  if (num + 1) % 5 == 0:
    print("")
```

We plot the correlation matrix.

```
fig, ax = plt.subplots(figsize=(25,20))
sns.set(font_scale=1.4)
sns.heatmap(data.corr(), annot=True, fmt=".2f", ax=ax, cmap="Blues")
plt.show()
sns.set(font_scale=1.0)
plt.savefig('images/with_gcs/feature_correlations.png')
plt.close()
```

Plot relative feature importances.

```
lr = LogisticRegression()
lr.fit(data, target)
feature_importance = abs(lr.coef_[0])
feature_importance = 100.0 * (feature_importance / feature_importance.max())
sorted_idx = np.argsort(feature_importance)
pos = np.arange(sorted_idx.shape[0]) + .5
featfig = plt.figure(figsize=(16,12))
featax = featfig.add_subplot(1, 1, 1)
featax.barh(pos, feature_importance[sorted_idx], align='center')
featax.set_yticks(pos)
featax.set_yticklabels(np.array(data.columns)[sorted_idx], fontsize=10)
featax.set_xlabel('Relative Feature Importance')
plt.savefig('images/with_gcs/relative_feature_importances.png')
plt.close()
```

Fit models and cross-validate

We proceed to fitting and evaluating a logistic regression model. We will adjust the regularization coefficient C and the class weight w by using (black-box) Bayesian optimization.

```
from sklearn.model_selection import cross_validate
def lrcv(C, w):
    scoring = ['roc_auc']
    scores = cross_validate(LogisticRegression(C=C,  class_weight={0:1,1:w}),
                            data,
                            target,
                            scoring=scoring,
                            cv=RepeatedStratifiedKFold(n_splits=5, n_repeats=10))
    val = scores['test_roc_auc'].mean()
    return val
from bayes_opt import BayesianOptimization
gp_params = {"alpha": 1e-5}
BO = BayesianOptimization(lrcv, {'C': (0.01,10), 'w': (1,1)})
BO.maximize(n_iter=20, **gp_params) # Try increasing the n_iter parameter to run the optimization longer.
print('-' * 53)
print('Final Results')
print('Classifier: %f' % BO.res['max']['max_val'])
C = BO.res['max']['max_params']['C']
w = BO.res['max']['max_params']['w']
lr = LogisticRegression(C=C, class_weight={0:1,1:w})
print(BO.res['max']['max_params'])
scoring = ['roc_auc', 'precision', 'recall', 'f1']
scores = cross_validate(lr, data, target, scoring=scoring, cv=RepeatedStratifiedKFold(n_splits=5, n_repeats=10),
return_train_score=True)
print('Averages of training scores:')
print('rocauc\tpreci\trecall\tf1')
print('{roc_auc:0.2f}\t{precision:0.2f}\t{recall:0.2f}\t{f1:0.2f}'.format(roc_auc=scores['train_roc_auc'].mean(),
                                                                           precision=scores['train_precision'].mean(),
                                                                           recall=scores['train_recall'].mean(),
                                                                           f1=scores['train_f1'].mean()))
print('Averages of testing scores:')
print('rocauc\tpreci\trecall\tf1')
print('{roc_auc:0.2f}\t{precision:0.2f}\t{recall:0.2f}\t{f1:0.2f}'.format(roc_auc=scores['test_roc_auc'].mean(),
                                                                           precision=scores['test_precision'].mean(),
                                                                           recall=scores['test_recall'].mean(),
                                                                           f1=scores['test_f1'].mean()))
```

Prediction on the full dataset

We illustrate the model performance by making predictions of the full dataset. In order to truthfully report the performance we use "cross-validated predictions". Here, for each data point the prediction is obtained from a model that was fitted without using this point.

```
from sklearn.model_selection import cross_val_predict, LeaveOneOut
pred_proba = cross_val_predict(lr, data, target, cv=LeaveOneOut(), method='predict_proba')
```

We store the outcome in a dataframe and plot both a normalized histogram and a (estimated) continuous distribution for survived and deceased separately.

```
results = pd.DataFrame()
results['true'] = target
#results['true'] = target_selected
results['pred_proba'] = pred_proba[:,1]
plt.figure(figsize=(15,5))
plt.title("Prediction probabilites survived & deceased (normalized histogram)")
plt.ylabel("Density")
sns.distplot(results[results['true'] == 1]['pred_proba'], bins=10, kde=False, label='deceased', norm_hist=True)
sns.distplot(results[results['true'] == 0]['pred_proba'], bins=10, kde=False, label='survived', norm_hist=True)
plt.xlabel("Predicted probability")
plt.legend()
plt.figure(figsize=(15,5))
plt.title("Prediction probabilites survived & deceased (kde)")
plt.ylabel("Density")
sns.distplot(results[results['true'] == 1]['pred_proba'], bins=10, hist=False, label='deceased')
sns.distplot(results[results['true'] == 0]['pred_proba'], bins=10, hist=False, label='survived')
plt.xlabel("Predicted probability")
plt.legend()
```

In order to make strict classifications and compute the accuracy, we set a threshold.

```
threshold = 0.5
results['pred'] = 1 * (results['pred_proba'] > threshold)
```

The misclassifications are examined below.

```
results[results['true'] != results['pred']].to_csv('images/with_gcs/misclassified.csv')
from sklearn.metrics import confusion_matrix, accuracy_score
print('Accuracy: {}%'.format(np.round(100 * accuracy_score(results['true'], results['pred']), 2)))
print()
print('Confusion matrix:')
cm = confusion_matrix(results['true'], results['pred'])
print(cm)
print()
print("False positives: {}".format(cm[0][1]))
print("False negatives: {}".format(cm[1][0]))
```

Calculate features for each time window (multiprocess)

```
from sklearn.preprocessing import StandardScaler
# Calculate features for the full dataset
data_full, target = prepare_data(df_patients, df_icp, df_map, df_cpp, df_mr, df_er)
# Scale the features
scaler = StandardScaler()
data_scaled = pd.DataFrame(data=scaler.fit_transform(data_full), index=data_full.index, columns=data_full.columns)
# Sort indices
data = data_scaled.sort_index()
target = target.sort_index()
# Create dictionaries for the features
data_full_pred = dict()  # All the features computed from truncated time series
data_pred = dict()  # All the scaled features
import multiprocessing as mp
hours = range(24, 128, 8)
manager = mp.Manager()
data_full_pred = manager.dict()
n_cores = 4
```

```
# created pool running maximum 4 cores
pool = mp.Pool(n_cores)
# Execute the feature calculation in parallel
for hour in hours:
    pool.apply_async(multiprocess_prepare_data, args=(df_patients,
                                                    df_icp[df_icp.delta < str(hour) + "h"],
                                                    df_map[df_map.delta < str(hour) + "h"],
                                                    df_cpp[df_cpp.delta < str(hour) + "h"],
                                                    df_mr[df_mr.delta < str(hour) + "h"],
                                                    df_er[df_er.delta < str(hour) + "h"],
                                                    data_full_pred,
                                                    hour))
# Tell the pool that there are no more tasks to come and join
pool.close()
pool.join()
for hour in hours:
  data_pred[hour] = pd.DataFrame(data=scaler.transform(data_full_pred[hour]), index=data_full_pred[hour].index,
columns=data_full_pred[hour].columns)
  data_pred[hour] = data_pred[hour].sort_index()
```

Calculate cross-validated AUC-ROC

```
from sklearn.model_selection import KFold, StratifiedKFold, RepeatedStratifiedKFold
from sklearn.metrics import roc_curve, auc, roc_auc_score
from scipy import interp
fold_method = RepeatedStratifiedKFold(n_splits=5, n_repeats=20)
lr_model = LogisticRegression(C=C, class_weight={0:1,1:w})
aucs_train = dict()
aucs_test = dict()
for hours in range(24, 128, 8):
  aucs_train[hours] = list()
  aucs_test[hours] = list()
selected_columns = data.columns[rfecv.support_]
data = data[selected_columns]

for train_indices, test_indices in fold_method.split(data, target):
  # train_indices and test_indices are positional indices, transforming them to patient_ids:
  train_patients, test_patients = data.index[train_indices], data.index[test_indices]

  # Train the fold specific model with full time series
  classifier = lr_model.fit(data.loc[train_patients], target.loc[train_patients])

  # Calculate metrics for each time window
  for hours in range(24, 128, 8):
    preds_train = classifier.predict_proba(data_pred[hours][selected_columns].loc[train_patients])[:,1]
    preds_test = classifier.predict_proba(data_pred[hours][selected_columns].loc[test_patients])[:,1]

    score_train = roc_auc_score(target.loc[train_patients], preds_train)
    score_test = roc_auc_score(target.loc[test_patients], preds_test)

    aucs_train[hours].append(score_train)
    aucs_test[hours].append(score_test)
```

Calculate hourly AUC means, AUC standard deviations and plot hourly AUCs with their error estimates

```
def auc_stats(aucs):
  aucs_mean = []
  aucs_std = []
  aucs_lower = []
  aucs_upper = []
  for hours in range(24, 128, 8):
    mean = np.mean(aucs[hours])
    aucs_mean.append(mean)
    std = np.std(aucs[hours])
    aucs_std.append(std)
```

```
    lower = mean - std
    aucs_lower.append(lower)
    upper = mean + std
    aucs_upper.append(upper)

  return aucs_mean, aucs_lower, aucs_upper
aucs_train_mean, aucs_train_lower, aucs_train_upper = auc_stats(aucs_train)
aucs_test_mean, aucs_test_lower, aucs_test_upper = auc_stats(aucs_test)
time = range(24, 128, 8)
plt.figure(figsize=(16,12))
plt.plot(time, aucs_train_mean, color='black', linestyle='--', label=r'AUROC $\pm$ 1 std. dev. (train)')
plt.plot(time, aucs_test_mean, color='red', label=r'AUROC $\pm$ 1 std. dev. (validation)')
plt.fill_between(time, aucs_train_lower, aucs_train_upper, color='black', alpha=.2)
plt.fill_between(time, aucs_test_lower, aucs_test_upper, color='red', alpha=.1)
plt.xlabel('Time (h)')
plt.ylabel('Area under ROC')
plt.legend(loc='upper right')
plt.ylim((0.6,1.0))
plt.savefig('images/with_gcs/auc_roc.png')
plt.show()
plt.close()
```

View the predictions dynamically

One of the central requirements for our model was to have it predict dynamically, i.e. to make it sensitive to changes in the ICP-MAP measurements for each patient. The features facilitating this are naturally the final 8h means of each derived series as well as their linear trend coefficients. Below we illustrate this by rolling out the monitor data in 8 hour windows and predicting as we go.

Notice that the model is fit on the full dataset of untruncated time series. While the rolled out monitor data is in principle unseen to the model, some features such as the initial 24h means do not change. One should therefore view this primarily as an illustration and not a test.

Computing the features for each 8h step takes a few minutes.

```
lr = LogisticRegression(C=C, class_weight={0:1,1:w})
lr.fit(data[selected_columns], target)
predict_proba = dict()
all_proba = pd.DataFrame() # a dataframe for the predictions
all_proba['true'] = target
for hours in tqdm_notebook(range(24, 128, 8), ncols=1000, desc="Hours"):
  predict_proba[hours] = lr.predict_proba(data_pred[hours][selected_columns])[:,1] # the slicing chooses the probability of death for each patient
  pred = pd.DataFrame(predict_proba[hours], index=data_pred[hours].index)
  all_proba[hours] = pred
proba_dead = all_proba[all_proba.true == 1].drop(columns='true').transpose().sort_index()
proba_alive = all_proba[all_proba.true == 0].drop(columns='true').transpose().sort_index()
df_icp['hours'] = df_icp['delta'].map(lambda x: x.total_seconds() / 60 / 60)
df_map['hours'] = df_map['delta'].map(lambda x: x.total_seconds() / 60 / 60)
df_cpp['hours'] = df_cpp['delta'].map(lambda x: x.total_seconds() / 60 / 60)
df_mr['hours'] = df_mr['delta'].map(lambda x: x.total_seconds() / 60 / 60)
df_er['hours'] = df_er['delta'].map(lambda x: x.total_seconds() / 60 / 60)
```

It is interesting to view how the predictions evolve for patients who deceased. We plot the predictions on top of their ICP-MAP-CPP data.

```
for i in proba_dead.columns:
  fig, ax1 = plt.subplots()

  fig.set_size_inches(25, 5)
  plt.title('Patient {} deceased'.format(i))
  plt.xlabel("Time passed (hours)")
  p0, = ax1.plot((proba_dead.index), proba_dead[i], label="estimate", color='red')
  ax1.set_ylim([0,1.01])
  ax1.set_ylabel('Estimate', fontsize=12)
  ax1.tick_params('y', colors='red')
  ax1.grid(visible=False)
```

```
  ax2 = ax1.twinx()
  p1, = ax2.plot(df_icp[df_icp.id == i].hours, df_icp[df_icp.id == i].value, label='icp')
  p2, = ax2.plot(df_map[df_map.id == i].hours, df_map[df_map.id == i].value, label='map')
  p3, = ax2.plot(df_cpp[df_cpp.id == i].hours, df_cpp[df_cpp.id == i].value, label='cpp')
  ax2.set_ylim([0, 150])
  ax2.set_ylabel('mmHg', fontsize=12)

  ax3 = ax1.twinx()
  ax3.spines["right"].set_position(("axes", 1.03))
  p4, = ax3.plot(df_er[df_er.id == i].hours, df_er[df_er.id == i].value, label='er', color='black', marker='o', linestyle=':')
  p5, = ax3.plot(df_mr[df_mr.id == i].hours, df_mr[df_mr.id == i].value, label='mr', color='magenta', marker='x',
linestyle=':')
  ax3.set_ylim([0.9,6.1])
  ax3.set_ylabel('Eye/Motor response', fontsize=12)

  lines = [p0, p1, p2, p3, p4, p5]

  ax3.legend(lines, [l.get_label() for l in lines], loc=2)

  fig.tight_layout()
  fig.savefig('images/with_gcs/deceased/' + str(i) + '_timeseries.png')
  plt.close()
for i in proba_alive.columns:
  fig, ax1 = plt.subplots()

  fig.set_size_inches(25, 5)
  plt.title('Patient {} survived'.format(i))
  plt.xlabel("Time passed (hours)")
  p0, = ax1.plot((proba_alive.index), proba_alive[i], label="estimate", color='red')
  ax1.set_ylim([0,1.01])
  ax1.set_ylabel('Estimate', fontsize=12)
  ax1.tick_params('y', colors='red')
  ax1.grid(visible=False)

  ax2 = ax1.twinx()
  p1, = ax2.plot(df_icp[df_icp.id == i].hours, df_icp[df_icp.id == i].value, label='icp')
  p2, = ax2.plot(df_map[df_map.id == i].hours, df_map[df_map.id == i].value, label='map')
  p3, = ax2.plot(df_cpp[df_cpp.id == i].hours, df_cpp[df_cpp.id == i].value, label='cpp')
  ax2.set_ylim([0, 150])
  ax2.set_ylabel('mmHg', fontsize=12)

  ax3 = ax1.twinx()
  ax3.spines["right"].set_position(("axes", 1.03))
  p4, = ax3.plot(df_er[df_er.id == i].hours, df_er[df_er.id == i].value, label='er', color='black', marker='o', linestyle=':')
  p5, = ax3.plot(df_mr[df_mr.id == i].hours, df_mr[df_mr.id == i].value, label='mr', color='magenta', marker='x',
linestyle=':')
  ax3.set_ylim([0.9,6.1])
  ax3.set_ylabel('Eye/Motor response', fontsize=12)

  lines = [p0, p1, p2, p3, p4, p5]

  ax3.legend(lines, [l.get_label() for l in lines], loc=2)

  fig.tight_layout()
  fig.savefig('images/with_gcs/survived/' + str(i) + '_timeseries.png')
  plt.close()
```

Let's take a closer look at our model.

```
from matplotlib.ticker import FixedFormatter
i = np.random.choice(proba_dead.columns)
importance_dict = {}
for hour in proba_dead.index:
  importance_dict[hour] = data_pred[hour][selected_columns].loc[i] * lr.coef_[0]
plt.figure(figsize=(25,5))
sns.heatmap(np.array([importance_dict[hour] for hour in proba_dead.index]).T,
```

```
          linewidth=0.1,
          cmap=sns.color_palette("coolwarm", 6),
          #cmap=sns.cubehelix_palette(n_colors=6, as_cmap=False),
          #cbar_kws={'label': 'indication', 'ticks': [-0.82,-0.5,-0.16,0.16,0.5,0.82], 'format': FixedFormatter(['extremely good','good','slightly good','slightly bad','bad','extremely bad'])},
          #vmin=-1, vmax=1,
          #cbar_kws={'label': 'indication', 'ticks': [-1.65,-1.0,-0.35,0.35,1.0,1.65], 'format': FixedFormatter(['extremely good','good','slightly good','slightly bad','bad','extremely bad'])},
          #vmin=-2, vmax=2,
          #cbar_kws={'label': 'indication', 'ticks': [-1.65,-1.0,-0.35,0.35,1.0,1.65], 'format': FixedFormatter(['extremely good','good','slightly good','slightly bad','bad','extremely bad'])},
          #vmin=-2, vmax=2,
          cbar_kws={'label': 'indication', 'ticks': [-2.5,-1.5,-0.5,0.5,1.5,2.5], 'format': FixedFormatter(['extremely good','good','slightly good','slightly bad','bad','extremely bad'])},
          vmin=-3, vmax=3,
          #cbar_kws={'label': 'indication', 'ticks': [-3.3,-2.0,-0.7,0.7,2.0,3.3], 'format': FixedFormatter(['extremely good','good','slightly good','slightly bad','bad','extremely bad'])},
          #vmin=-4, vmax=4,
          #cbar_kws={'label': 'indication', 'ticks': [-4.1,-2.5,-0.8,0.8,2.5,4.1], 'format': FixedFormatter(['extremely good','good','slightly good','slightly bad','bad','extremely bad'])},
          #vmin=-5, vmax=5,
          xticklabels=[str(hour) + ': ' + str(int(np.rint(100 * proba_dead[i][hour]))) + '%' for hour in proba_dead.index],
          yticklabels=importance_dict[24].keys())
plt.title('Patient {} deceased'.format(i))
plt.xlabel('Time passed (hours): Estimated probability of death')
from matplotlib.ticker import FixedFormatter
for i in proba_dead.columns:
  importance_dict = {}
  for hour in proba_dead.index:
    importance_dict[hour] = data_pred[hour][selected_columns].loc[i] * lr.coef_[0]
  plt.figure(figsize=(25,5))
  sns.heatmap(np.array([importance_dict[hour] for hour in proba_dead.index]).T,
            linewidth=0.1,
            cmap=sns.color_palette("coolwarm", 6),
            #cmap=sns.cubehelix_palette(n_colors=6, as_cmap=False),
            #cbar_kws={'label': 'indication', 'ticks': [-0.82,-0.5,-0.16,0.16,0.5,0.82], 'format': FixedFormatter(['extremely good','good','slightly good','slightly bad','bad','extremely bad'])},
            #vmin=-1, vmax=1,
            #cbar_kws={'label': 'indication', 'ticks': [-1.65,-1.0,-0.35,0.35,1.0,1.65], 'format': FixedFormatter(['extremely good','good','slightly good','slightly bad','bad','extremely bad'])},
            #vmin=-2, vmax=2,
            #cbar_kws={'label': 'indication', 'ticks': [-1.65,-1.0,-0.35,0.35,1.0,1.65], 'format': FixedFormatter(['extremely good','good','slightly good','slightly bad','bad','extremely bad'])},
            #vmin=-2, vmax=2,
            cbar_kws={'label': 'indication', 'ticks': [-2.5,-1.5,-0.5,0.5,1.5,2.5], 'format': FixedFormatter(['extremely good','good','slightly good','slightly bad','bad','extremely bad'])},
            vmin=-3, vmax=3,
            #cbar_kws={'label': 'indication', 'ticks': [-3.3,-2.0,-0.7,0.7,2.0,3.3], 'format': FixedFormatter(['extremely good','good','slightly good','slightly bad','bad','extremely bad'])},
            #vmin=-4, vmax=4,
            #cbar_kws={'label': 'indication', 'ticks': [-4.1,-2.5,-0.8,0.8,2.5,4.1], 'format': FixedFormatter(['extremely good','good','slightly good','slightly bad','bad','extremely bad'])},
            #vmin=-5, vmax=5,
            xticklabels=[str(hour) + ': ' + str(int(np.rint(100 * proba_dead[i][hour]))) + '%' for hour in proba_dead.index],
            yticklabels=importance_dict[24].keys())
  plt.title('Patient {} deceased'.format(i))
  plt.xlabel('Time passed (hours): Estimated probability of death')
  plt.savefig('images/with_gcs/deceased/' + str(i) + '_featureimportance.png')
  plt.close()
from matplotlib.ticker import FixedFormatter
for i in proba_alive.columns:
  importance_dict = {}
  for hour in proba_alive.index:
    importance_dict[hour] = data_pred[hour][selected_columns].loc[i] * lr.coef_[0]
  plt.figure(figsize=(25,5))
  sns.heatmap(np.array([importance_dict[hour] for hour in proba_alive.index]).T,
```

```
            linewidth=0.1,
            cmap=sns.color_palette("coolwarm", 6),
            #cmap=sns.cubehelix_palette(n_colors=6, as_cmap=False),
            #cbar_kws={'label': 'indication', 'ticks': [-0.82,-0.5,-0.16,0.16,0.5,0.82], 'format': FixedFormatter(['extremely
good','good','slightly good','slightly bad','bad','extremely bad'])},
            #vmin=-1, vmax=1,
            #cbar_kws={'label': 'indication', 'ticks': [-1.65,-1.0,-0.35,0.35,1.0,1.65], 'format': FixedFormatter(['extremely
good','good','slightly good','slightly bad','bad','extremely bad'])},
            #vmin=-2, vmax=2,
            #cbar_kws={'label': 'indication', 'ticks': [-1.65,-1.0,-0.35,0.35,1.0,1.65], 'format': FixedFormatter(['extremely
good','good','slightly good','slightly bad','bad','extremely bad'])},
            #vmin=-2, vmax=2,
            cbar_kws={'label': 'indication', 'ticks': [-2.5,-1.5,-0.5,0.5,1.5,2.5], 'format': FixedFormatter(['extremely
good','good','slightly good','slightly bad','bad','extremely bad'])},
            vmin=-3, vmax=3,
            #cbar_kws={'label': 'indication', 'ticks': [-3.3,-2.0,-0.7,0.7,2.0,3.3], 'format': FixedFormatter(['extremely
good','good','slightly good','slightly bad','bad','extremely bad'])},
            #vmin=-4, vmax=4,
            #cbar_kws={'label': 'indication', 'ticks': [-4.1,-2.5,-0.8,0.8,2.5,4.1], 'format': FixedFormatter(['extremely
good','good','slightly good','slightly bad','bad','extremely bad'])},
            #vmin=-5, vmax=5,
            xticklabels=[str(hour) + ': ' + str(int(np.rint(100 * proba_alive[i][hour]))) + '%' for hour in proba_alive.index],
            yticklabels=importance_dict[24].keys())
  plt.title('Patient {} survived'.format(i))
  plt.xlabel('Time passed (hours): Estimated probability of death')
  plt.savefig('images/with_gcs/survived/' + str(i) + '_featureimportance.png')
  plt.close()
```

Model feature importances

```
print('Feature coefficients:')
dict(zip(data.columns, np.round(lr.coef_[0],2)))
plt.figure()
plt.title('Predictive role of (high numerical values of) features.')
#ticks = [-0.9,-0.3,0.3,0.9]
#vmin = -1.2
#vmax = 1.2
ticks = [-1.2,-0.4,0.4,1.2]
vmin = -1.6
vmax = 1.6
sns.heatmap(np.array([lr.coef_[0]]).T,
           linewidth=0.1,
           cmap=sns.color_palette("coolwarm", 4),
           cbar_kws={'label': 'predictor', 'ticks': ticks, 'format': FixedFormatter(['good','slightly good','slightly
bad','bad',])},
           vmin=vmin, vmax=vmax,
           xticklabels=[],
           yticklabels=importance_dict[24].keys(),
           square=True)
plt.savefig('images/with_gcs/overall_feature_importances.png')
plt.close()
```

Evolution of prediction distribution over time

```
all_proba_plot = all_proba.copy()
while all_proba_plot.iloc[0]['true'] == 0:
  all_proba_plot = all_proba_plot.sample(frac=1)
fig = plt.figure(figsize=(25,10))
fig.suptitle('Predicted scores for survived / deceased by hour.')
plot_data_1 = pd.melt(all_proba_plot, id_vars=['true'], value_vars=[24, 32, 40, 48, 56, 64], var_name='hour',
value_name='score')
plot_data_1['true'] = plot_data_1['true'].map({0.0: 'survived', 1.0: 'deceased'})
plot_data_2 = pd.melt(all_proba_plot, id_vars=['true'], value_vars=[72, 80, 88, 96, 104, 112, 120], var_name='hour',
value_name='score')
plot_data_2['true'] = plot_data_2['true'].map({0.0: 'survived', 1.0: 'deceased'})
plt.subplot(2,1,1)
```

```
sns.violinplot(x="hour", y="score", hue="true", cut=0, scale='count', bw=0.2, data=plot_data_1, split=True)
plt.subplot(2,1,2)
sns.violinplot(x="hour", y="score", hue="true", cut=0, scale='count', bw=0.2, data=plot_data_2, split=True)
plt.savefig('images/with_gcs/violinplot.png')
plt.close()
```

Copy all images to Google Cloud Bucket

```
!gsutil -m cp -r images/with_gcs/ gs://hus-aip-tbi/images/
```
